# Functional roles of the membrane-associated AAV protein MAAP

**DOI:** 10.1038/s41598-021-01220-7

**Published:** 2021-11-04

**Authors:** Lionel Galibert, Amira Hyvönen, Reetta A. E. Eriksson, Salla Mattola, Vesa Aho, Sami Salminen, Justin D. Albers, Sanna K. Peltola, Saija Weman, Tiina Nieminen, Seppo Ylä-Herttuala, Hanna P. Lesch, Maija Vihinen-Ranta, Kari J. Airenne

**Affiliations:** 1 Kuopio Center for Gene and Cell Therapy, Kuopio, Finland; 2 FinVector, Kuopio, Finland; 3 Department of Biological and Environmental Science and Nanoscience Center, University of Jyvaskyla, Jyväskylä, Finland; 4 A.I. Virtanen Institute for Molecular Sciences, University of Eastern Finland, Kuopio, Finland; 5 Gene Therapy Unit and Research Center, Kuopio University Hospital, Kuopio, Finland

**Keywords:** Gene therapy, Molecular engineering

## Abstract

With a limited coding capacity of 4.7 kb, adeno-associated virus (AAV) genome has evolved over-lapping genes to maximise the usage of its genome. An example is the recently found ORF in the *cap* gene, encoding membrane-associated accessory protein (MAAP), located in the same genomic region as the VP1/2 unique domain, but in a different reading frame. This 13 KDa protein, unique to the dependovirus genus, is not homologous to any known protein. Our studies confirm that MAAP translation initiates from the first CTG codon found in the VP1 ORF2. We have further observed MAAP localised in the plasma membrane, in the membranous structures in close proximity to the nucleus and to the nuclear envelope by co-transfecting with plasmids encoding the wild-type AAV (wt-AAV) genome and adenovirus (Ad) helper genes. While keeping VP1/2 protein sequence identical, both inactivation and truncation of MAAP translation affected the emergence and intracellular distribution of the AAV capsid proteins. We have demonstrated that MAAP facilitates AAV replication and has a role in controlling Ad infection. Additionally, we were able to improve virus production and capsid integrity through a C-terminal truncation of MAAP while other modifications led to increased packaging of contaminating, non-viral DNA. Our results show that MAAP plays a significant role in AAV infection, with profound implications for the production of therapeutic AAV vectors.

## Introduction

Adeno-associated virus (AAV) serotype 2 is a dependoparvovirus with a ssDNA genome of 4679 bases. Recombinant AAV vectors have gained interest for their applications in gene therapy. In recent years, the FDA has approved two AAV therapeutic applications, Luxturna for the treatment of Leber’s congenital amaurosis and Zolgensma to treat spinal muscular atrophy.

AAV replication is dependent on helper viruses such as adenoviruses (Ad)^1^ or herpesviruses^2^. The AAV2 genome is flanked at both ends by 145-base T-shaped structures known as inverted terminal repeats (ITRs) which are necessarily involved in AAV’s genome replication, second-strand synthesis^3,4^, encapsidation^5^, and genomic integration^6,7^.

Replication is mediated by the large Rep proteins, Rep78 and 68^8,9^ while the small Rep proteins, Rep52 and Rep40, are required for the packaging of the single-stranded genome^5,10^ into preformed empty capsids^11^. Indeed, space within the AAV genome is so efficiently used that alternative splicing and non-canonical start codons in the *cap* gene encode three capsid proteins, VP1, VP2 and VP3 (VPs), from the three overlapping reading frames^12,13^, as well as the Assembly Activating Protein (AAP) which is transcribed as the result of a frame-shift in the VP2/3 reading frame^14^. AAV2 AAP’s primary role is to target the VPs to the nucleolus but it is also frequently involved in capsid assembly. Other reports have shown that AAP is not strictly required for the capsid assembly of some AAV serotypes such as AAV4, 5, 11 and rh32.33^15,16^. The icosahedral capsid of AAV is composed of 60 assembled VP proteins and although serotypes vary, generally VP3 dominates the structure at a ratio of 10:1:1 with the VP1 and VP2 evenly comprising the balance^17^. Studies of Hela cells co-infected with wt-Ad and wt-AAV suggest that the well-known phenomenon of AAV subcellular compartmentalisation is the result of the capsid assembly being initiated in the nucleolus while the DNA packaging likely occurs separately, in the nucleoplasm^18^. At later stages, the Rep proteins are enriched at the nuclear periphery. Assembled AAV capsids of different serotypes co-localise with AAP either in the nucleus, the nucleolus, or are clustered around the nuclear membrane^5^.

Recently, through systemic mutagenesis performed in the *cap* ORF, Ogden and co-workers^19^ identified a second ORF nested in the VP1/2 unique domain within a different reading frame, encoding a new viral protein. C-terminal fusion of the protein to Green Fluorescent Protein (GFP) or to Flag-tag revealed an association with cell membranes, leading the authors to name it “membrane-associated accessory protein” (MAAP). Recombinant viruses produced with inactivated MAAP were viable and produced similar titers as AAVs encoding the wt-MAAP. However, unless MAAP was supplied *in trans,* MAAP-mutated AAVs were outcompeted by viruses encoding wt-*cap*. Based on these results, Ogden and collaborators proposed that MAAP may be involved in competitive exclusion between different serotype AAV genomes. This finding is parsimonious with capsid and genome couplings observed in the generation of engineered AAV capsid libraries^20^.

Here we report confirmation that the first CTG (leucine) codon of the *MAAP* ORF is indeed the start codon for MAAP translation. Mutating the start codon or introducing stop codons along the *MAAP* coding sequence led to the absence of MAAP or production of a C-terminally truncated form in similar or reduced levels, when compared to wt-MAAP. Confocal imaging and analyses demonstrated that in transfected cells MAAP localises to plasma membrane, perinuclear membranous structures, and to the nuclear envelope. The inactivation and truncation of MAAP affected the production and intracellular distribution of AAV capsid proteins, suggesting that MAAP may play an important role in infection. While wt-AAV2 resulted in higher viral genomic (vg) titers at 24 h post transfection (hpt), at 72 hpt, most of the MAAP-modified viruses produced titers exceeding those of wt-AAV2. AAVs encoding C-terminally truncated MAAP or having no detectable MAAP also showed better capsid integrity than the wild-type virus. Compared to natural AAV2 and Ad co-infection, MAAP-modified viruses replicated at a lower speed than wt-AAV2, suggesting that MAAP is an infection-accelerating factor for AAV. These results extend and bring new information on the novel AAV-encoded protein MAAP and have important practical implications on how to improve AAV generation for gene therapy applications.

## Results

### MAAP translation initiates at the first CTG codon of the VP1 ORF2

**Figure 1:**
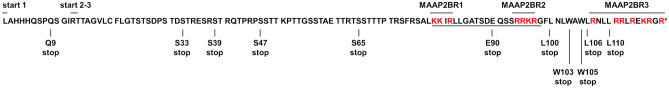
MAAP sequence. MAAP is encoded in the wt-AAV2 genome (GenBank Sequence ID: AF043303.1) at nucleotides 2282-2641. The ORF is located on the same DNA region encoding the VP1/2 unique domains. VP1/2 are encoded on reading frame + 1 and MAAP on reading frame + 2. MAAP translation initiates at a CTG codon (start 1), encoding a leucine which corresponds in position to VP1 proline in position 27. The next potential start codons are MAAP-T13 (ACG) and MAAP-R14 (AGG) (start 2-3). In red are the three basic-amino-acid-rich clusters MAAP2BR1, MAAP2BR2 and MAAP2BR3. Underlined is the sequence of the peptide used to generate MAAP polyclonal antiserum. All studied amino acid substitutions are shown below the sequence.

Analysis of the MAAP sequence to identify potential non-ATG initiation codons revealed that at least three different triplets could be used to initiate MAAP translation. These codons differ in one base from the canonical ATG start codon and can initiate translation^21^. The first CTG encountered on the MAAP reading frame was described as the principal translation initiation codon. It encodes a Leucine in the MAAP (MAAP-L1) and corresponds, in position, to VP1-P27. The following potential translation initiation codons were AGG (MAAP-R13) and ACG (MAAP-T14). An overview of the MAAP sequence along with the mutants that we created and studied is presented in Fig. 1, with the detailed mutations in Supplementary Table 1. Interestingly, the MAAP C-terminus contains three basic-amino-acid-rich (BR) clusters, KKIR (MAAP2BR1), RRKR (MAAP2BR2) and RNLLRRLREKRGR (MAAP2BR3) that could be involved in the cellular localisation of MAAP. Indeed, similar BR clusters were shown to act as nuclear localisation signal (NLS) for AAP^22^.

**Figure 2:**
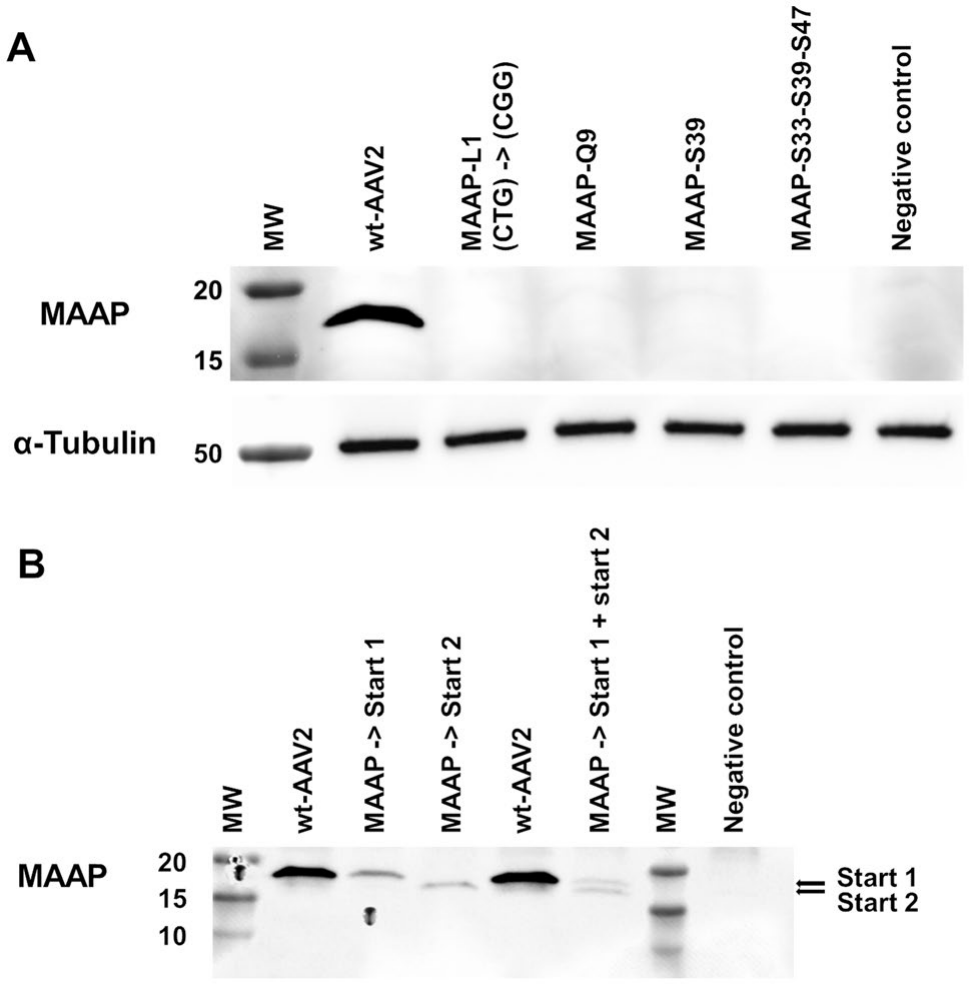
MAAP start codon identification and MAAP inactivation. (**A**) MAAP detected by immunoblot from 293 T cell lysates 24 hpt with wt- or MAAP mutants and Ad helper plasmids. Immunoblots of MAAP (top panel) and of α-Tubulin (lower panel) for equal sample loading. Left to right: (1) MW. (2) wt-AAV2. (3) AAV2 with MAAP-L1 (CTG) start codon modified to R1 (CGG). (4) MAAP-Q9. (5) MAAP-S39. (6) MAAP-S33-S39-S47. (7) non-transfected 293 T cells. (**B**) Start codon usage for MAAP translation. MAAP expressed from the first or second potential start codon and detected by immunoblot. Left to right: (1) MW. (2) wt-AAV2. (3) MAAP expressed from L1 (CTG) mutated to M (ATG). (4) MAAP expressed from R13 (AGG) mutated to M (ATG). (5) wt-AAV2. (6) v/v mix of MAAP expressed from L1 (CTG) mutated to M (ATG) and MAAP expressed from R13 (AGG) mutated to M (ATG). (7) MW. (8) non-transfected 293 T cells.

To analyse the start codon usage in the context of the wt-AAV2 genome, we mutated the MAAP-L1 (CTG) to MAAP-R1 (CGG) which disrupted the first potential start codon of MAAP (Fig. 2A). This resulted in our specific antibody raised against aa 79-98 of MAAP (Fig. 1, underlined) not detecting MAAP in immunoblotting. Similarly, introduction of a stop codon in place of MAAP-Q9, located between the first CTG and the second potential AGG start codon, also led to non-detection (Fig. 2A). Neither did we detect MAAP production when we introduced a stop codon in the place of MAAP-S39 nor when we placed three consecutive stop codons into MAAP-S33-S39-S47 (Fig. 2A). For the latter case, we cannot exclude the possible translation of a truncated N-terminally stable form of MAAP. In order to further characterise the size of MAAP translation initiation fragments we prepared plasmids encoding recombinant forms of MAAP. In one plasmid the MAAP-L1 CTG start codon was replaced by ATG. In the second plasmid we tested the second potential start codon, MAAP-R13, by modifying the AGG start codon to ATG. This produced an N-terminally truncated MAAP fragment (Fig. 2B). The size of the wt-MAAP corresponds to the recombinant MAAP, while the N-terminally truncated recombinant MAAP appeared at a lower molecular weight on the immunoblot.

We further interrogated the expression of MAAP by C-terminally fusing the *MAAP* ORF to *GFP* (*MAAP-GFP*) in the wt-AAV2 genome. This fusion resulted in functional disruption of VP1/2 proteins, due to the insertion of the *GFP* in the *cap* ORF frame + 2. However, the capacity to encode the AAP and VP3 proteins were expected to be retained. Thus, the fluorescence was expected to correlate with the transcription and translation activities of MAAP in the viral context. *MAAP-GFP* expression was compared to its variants in which stop codons were introduced in the 5’- end of the *MAAP* or with the mutated MAAP-L1 start codon. The experiments were conducted in the presence and absence of the Ad helper functions, even though the adenoviral E1 gene is already present in the 293 T cell genome^23^ (S1 Fig). A median fluorescence intensity (MFI) of 10,762 was measured for 293 T cells transfected with AAV2 plasmid encoding MAAP-GFP, whereas a co-transfection with Ad helper plasmid^24^ resulted in an almost threefold increase in MFI (30113). The results suggest active *MAAP-GFP* transcription and MAAP-GFP translation by the p40 promoter driven mRNA. All MAAP-GFP variants containing either mutated start codons or early stop codons showed about twofold lower MFI (16262) than the MAAP-GFP produced from wt-AAV2 genome (30113). This lowered production of mutated MAAP-GFPs is concordant with our prior immunoblotting results. None of the mutant MAAPs were observed in immunoblotting however GFP-mediated fluorescence was visible in all MAAP-GFP samples. The fact that 293 T cells produce Ad E1 A and B proteins^24,25^ may explain the observed basal GFP activity. On the other hand, a 1.8 fold higher MFI was observed in samples with Ad helper plasmid co-transfection. We did not detect MAAP production without GFP fusions and find it unlikely that stop codon read-through^26^ occurred, as no difference in MAAP-GFP variants production was observed when a single or multiple simultaneous stop codons were introduced into *MAAP-S33, -S39, or -S47*. These experiments further support that MAAP-L1 (CTG) is the primary MAAP translation start site.

### Kinetics of MAAP translation

**Figure 3:**
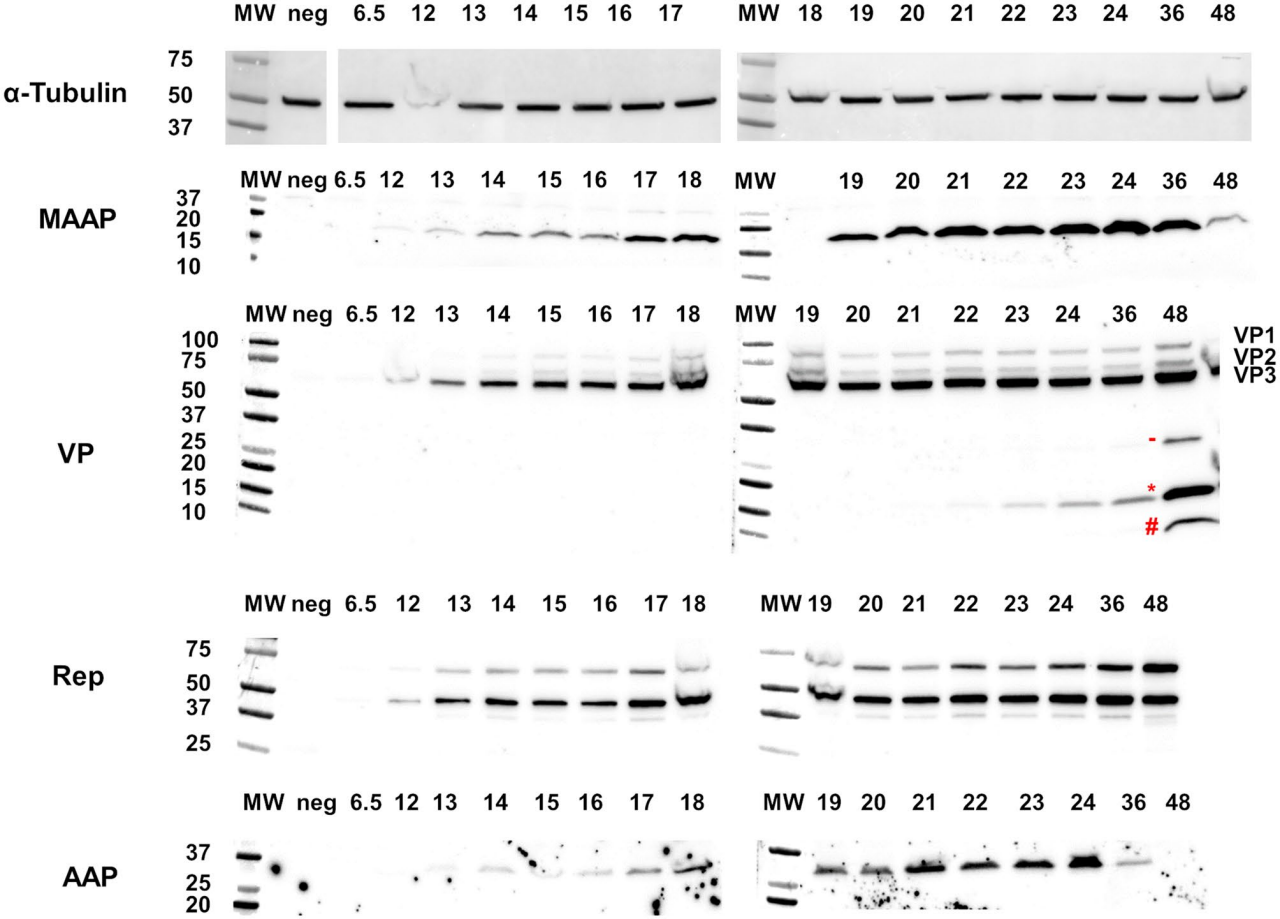
Kinetics of AAV2 protein production. Translation of AAV2 MAAP, VP, Rep, AAP and α-Tubulin studied by immunoblot from wt-AAV2 and Ad helper plasmid-transfected 293 T cells. From left to right: MW, non-transfected 293 T, wt-AAV2 cell lysates harvested from 6.5 to 48 hpt. Degradation products of VP proteins are annotated, the 32 kDa fragment (−), 18 kDa (*) and 12 kDa (#).

MAAP should be expressed from the *cap* gene, possibly from the spliced form of the p40 transcript encoding VP2/3 and AAP translation. According to ribosome scanning mechanism^21^, translation initiates at the CTG start codon of the MAAP (frame + 2), followed by VP2 translation at the ACG start codon (frame + 1), continuing with AAP translation at a CTG codon (frame + 2), and ending with the VP3 translation at an ATG codon (frame + 1). We studied the expression of the Rep78/52, VPs, AAP and MAAP proteins in 293 T cells during wt-AAV2 production (Fig. 3). Most of the proteins were clearly detectable at 12 hpt, with the exception of AAP at 13 hpt. Additionally, VP3 and Rep52 were very faintly detectable already at the 6.5 h time point. Protein production increased progressively and reached a plateau at 21 hpt. Capsid degradation was observed from 21 hpt on as the protein bands below VP3 on the immunoblot.

### Intracellular localisation of MAAP, capsid proteins and capsids in transfected cells

**Figure 4:**
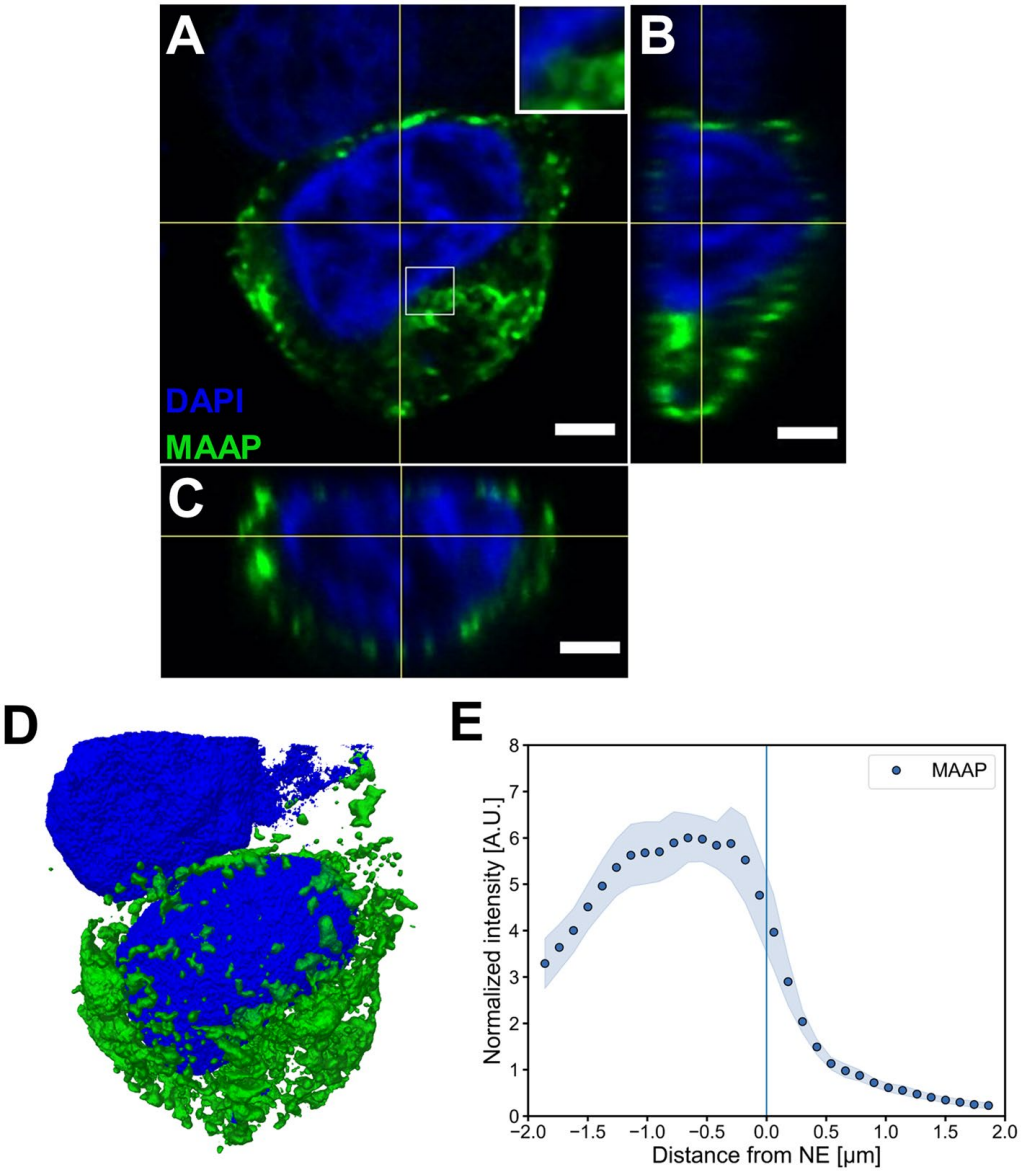
3D visualization of MAAP cellular distribution in transfected cells. 3D confocal analysis of representative cytoplasmic distribution of MAAP at 24 hpt in cells co-transfected with wt-AAV2 and Ad helper plasmids. (**A**) xy, (**B**) yz and (**C**) xz slices of confocal z-stack of cytoplasmic MAAP (green), and DAPI-stained cellular DNA (blue). Inset shows higher magnification image of the cytoplasmic MAAP (boxed area), scale bars, 3 μm. (**D**) 3D reconstruction of the nucleus and distribution of MAAP obtained by confocal microscopy. The MAAP appears in green, scale bar, 3 μm. See also S2 movie**. **(**E**) Quantitative analysis of MAAP distribution as function of distance from the nuclear envelope towards the nuclear centre, where nuclear envelope is located in 0.0. The 3D nuclear envelope boundary reconstruction was based on distribution of DAPI-labelled chromatin. The shaded areas around the data points represent the standard error of the mean (SEM, n = 6).

GFP-tagged MAAP was found associated with cellular membranes, specifically with the plasma membrane by Ogden and collaborators^19^. This is consistent with previously published studies predicting the presence of a membrane-binding motif, a C-terminal hydrophobic amphipathic α-helix, in MAAP^27^. Our studies sought to further characterise the specific subcellular distribution of MAAP in transfected 293 T cells and to reveal the effect of various mutations on the expression and distribution of MAAP, viral proteins and capsids. The 3D confocal imaging of anti-MAAP stained cells showed that at 24 hpt, with wt-AAV2 and Ad helper plasmids, MAAP was localised in the intracellular membranes, specifically in the plasma membrane and perinuclear ER (Fig. 4A–C). Furthermore, a proportion of MAAP was also found in clusters or small foci located in close proximity to the nuclear envelope (Fig. 4A). 3D reconstructions of the transfected cells showed that the size of the MAAP clusters varied largely. While most of MAAP accumulated in enlarged clusters located in the plasma membrane and in the perinuclear area, small foci were also detected in nuclear vicinity (Fig. 4D, S2 movie). A quantitative analysis of 3D confocal data as a function of distance from the nuclear envelope towards the nuclear centre or the cytoplasm confirmed that MAAP was distributed around the cytoplasm and the highest amount of MAAP was detected within some distance, 0.25–1.25 µm, from the nuclear envelope (Fig. 4E). Based on this analysis we cannot rule out that a small amount of MAAP could be located in the nucleus or to the nuclear envelope. This, however, can be an analysis artefact caused by inexact determination of the nuclear border due to diffraction-limited imaging and by the localisation of MAAP into nuclear invaginations as seen in the 3D rendering of the MAAP transfected cells. However, we cannot formally rule out that a small proportion of MAAP might be transported from the outer nuclear membrane to the inner nuclear membrane via the pore membrane across the nuclear pore complex (NPC)^28^. Further studies should therefore address whether MAAP is able to enter the nucleus.

**Figure 5:**
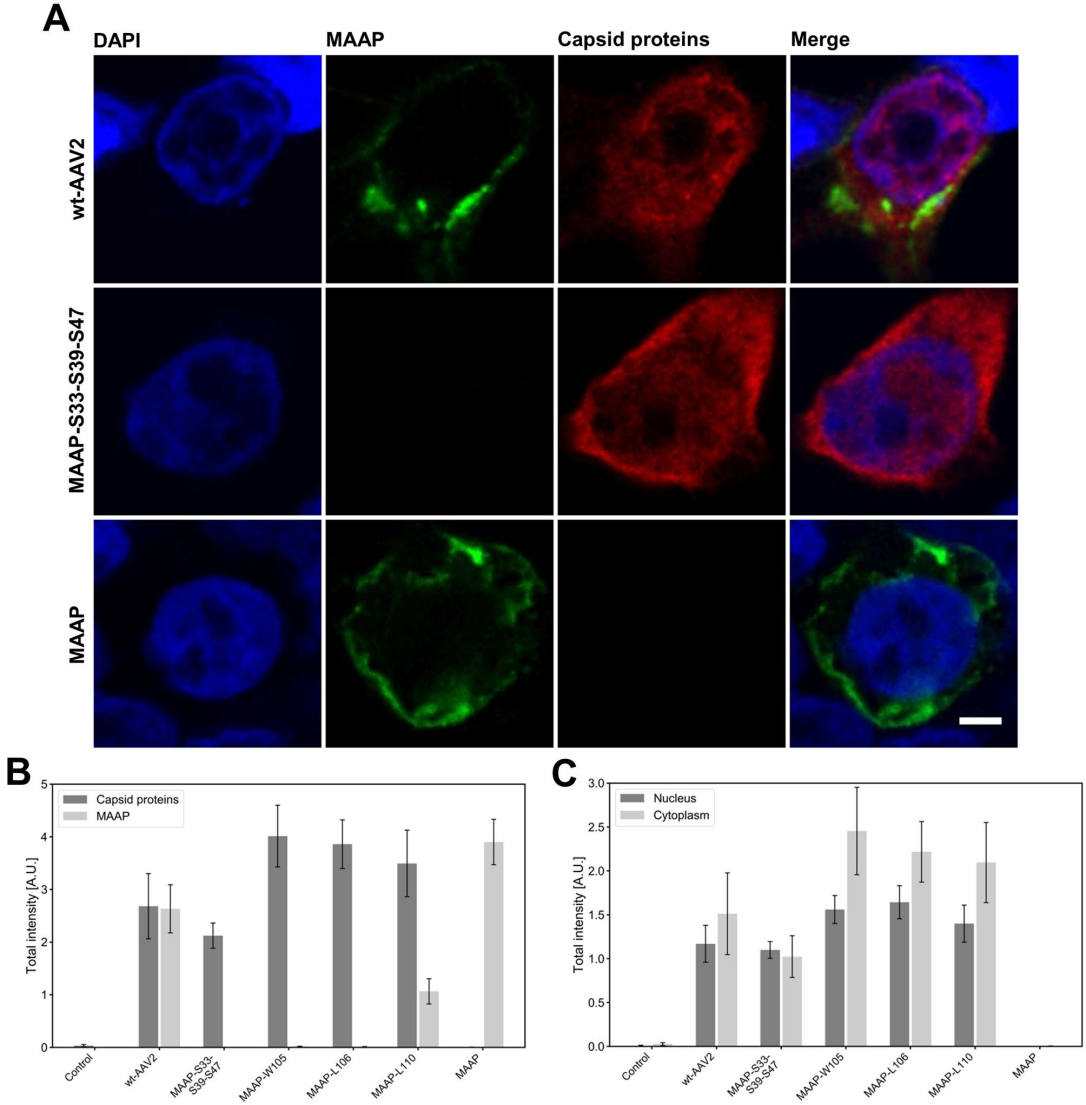
Localization and intensity of MAAP and AAV-2 capsid proteins in transfected cells. (**A**) Representative confocal images of intracellular localisation of MAAP and capsid proteins at 24 hpt. Cells were co-transfected with wt-AAV2 and MAAP-S33-S39-S47 variant together with Ad helper plasmid, or with recombinant MAAP expression plasmid without Ad helper plasmid. The cells were immunolabelled with polyclonal antibody against MAAP (green) and monoclonal antibody against viral capsid proteins VP1, VP2 and VP3 (red). Blue corresponds to DAPI staining. Scale bars, 3 µm. For analyses, the cells were either mock transfected (n = 19), co-transfected with wt-AAV2 (n = 11), MAAP-S33-S39-S47 (n = 19), MAAP-W105 (n = 20), MAAP-L106 (n = 17), MAAP-L110 (n = 20) together with Ad helper plasmid, or transfected with the recombinant MAAP plasmid (n = 18). (**B**) Quantitative analyses of the total intensities of MAAP (light grey) and capsid proteins (dark grey) at 24 hpt. (**C**) Quantitative analyses of the relative intensity of nuclear (dark grey) in comparison to cytoplasmic capsid proteins (light grey) at 24 hpt. For analysis, the cells were immunolabelled with antibodies against viral capsid protein VP1, VP2 and VP3 (red) and the nuclear boundary segmentation was based on distribution of DAPI-labelled chromatin (blue). The error bars show the SEM.

Comparison of MAAP expression in Ad helper co-transfected cells, either with wt-AAV2 or MAAP-deficient (MAAP-S33-S39-S47) plasmids, demonstrated that the modification of *MAAP* led to a loss of detectable MAAP when the antibody raised against the C-terminal end of MAAP was used for the detection. The expression of recombinant MAAP in the absence of Ad helper plasmid resulted in a similar MAAP distribution pattern in intracellular membranous structures than that seen in wt-AAV2 transfected cells (Fig. 5A). This similarity suggests that MAAP membrane targeting is independent of the expression of AAV2 proteins.

Using a quantitative analysis of confocal microscopy data, we further studied the emergence of MAAP in wt-AAV2 and recombinant MAAP expression plasmid transfected cells and changes in the amount of MAAP at 24 hpt. In accordance with the immunoblotting data, the results demonstrated that cells transfected with MAAP deficient mutants, MAAP-W105 and MAAP-L106, did not produce MAAP, while some was seen in cells transfected with MAAP-L110. Interestingly, transfections with these MAAP mutants resulted in a slightly increased level of capsid proteins in comparison to cells transfected with wt-AAV2 (Fig. 5B). As described above, confocal microscopy showed that MAAP was located in clusters of various sizes scattered throughout the plasma membrane, in structures we assume to be endoplasmic reticulum (ER), intracellular membranes and the nuclear envelope in transfected cells. To further identify the intracellular localisation of viral capsid proteins, and specifically the effect of MAAP mutations on these proteins, we analysed the cytoplasmic and nuclear distribution of capsid proteins in cells co-transfected with the wt-AAV2 or the MAAP mutant AAV2 plasmids together with the Ad helper plasmid at 24 hpt. As shown in Fig. 5C, the capsid proteins were located in the cytoplasm and the nucleoplasm. Comparison between wt-AAV2 and C-terminal MAAP mutant transfected cells showed that in both cases, the majority of the capsid proteins were located in the cytoplasm. However, transfection with MAAP-deficient AAV2 plasmid MAAP-S33-S39-S47 led to a decrease in total capsid protein intensity (AU) from 2.68 ± 0.62 observed in wt-AAV2 to 2.12 ± 0.24 (mean ± SEM), whereas the C-terminal mutants MAAP-105, -106 and -110 induced an increase in total intensity of capsid proteins, 4.01 ± 0.59, 3.86 ± 0.46 and 3.49 ± 0.63, respectively (mean ± SEM) (Fig. 5B). The analysis also indicated that the total amount of nuclear capsid proteins in wt-AAV2 and MAAP-mutant transfected cells was relatively similar; however, in the C-terminal mutant transfected cells, capsid proteins were slightly more enriched in the cytoplasm. In this regard, MAAP might play a potential role in the regulation of capsid protein expression or in the nuclear transport of capsid proteins.

**Figure 6:**
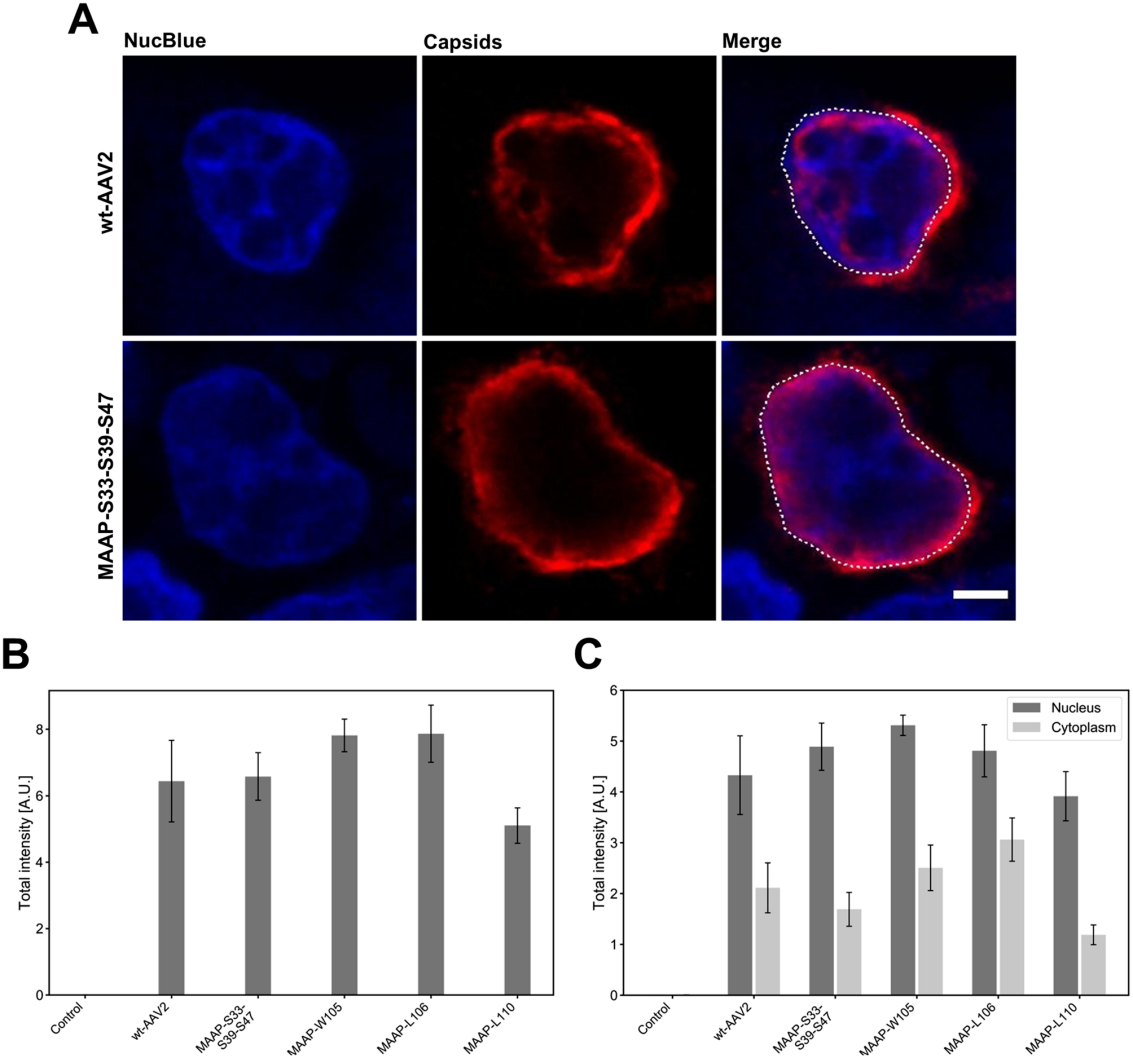
Intracellular distribution and intensity of AAV2 capsids in MAAP transfected cells. (**A**) Representative confocal images of intracellular localisation of capsids (red) in wt-AAV2 or MAAP-S33-S39-S47-transfected cells at 24 hpt**.** Cells were co-transfected with Ad helper plasmid. The cells were immunolabelled with an antibody against intact viral capsids (red). Blue corresponds to NucBlue chromatin staining. Nuclear border is indicated by dashed white line, scale bars, 3 µm. (**B**) Quantitative analyses of the total intensity of capsids in cells either mock transfected (n = 15) or co-transfected with wt-AAV2 (n = 12), MAAP-S33-S39-S47 (n = 12), MAAP-W105 (n = 11), MAAP-L106 (n = 13), MAAP-L110 (n = 7) together with Ad helper plasmid. (**C**) Analyses of the relative intensity of nuclear capsids (dark grey) in comparison to cytoplasmic capsids (light grey). For analyses, the cells were immunolabelled with antibodies against viral capsids (red) and the nuclear boundary segmentation was based on distribution of NucBlue-labelled chromatin (blue). Nuclear border is indicated by dashed white line. The error bars show the SEM.

To examine the role of MAAP in the intracellular distribution of AAV capsids, we observed the effect of MAAP mutations on the cellular emergence and localisation of capsids. Confocal imaging of wt-AAV2 and MAAP-deficient MAAP-S33-S39-S47-transfected cells revealed the presence of AAV capsids, detected with an antibody recognising the conformational epitope of intact AAV capsids, in the cytoplasm and nucleus (Fig. 6A). The quantitative analysis of capsid intensity demonstrated that capsids were produced in all cells transfected with either wt-AAV2 or MAAP-mutant constructs; however, transfection with one of the C-terminal mutants (MAAP-L110) led to a slight decrease in the total intensity of intact capsids (5.10 ± 0.53) compared to the total intact capsid intensity of wt-AAV transfection (6.44 ± 1.23) (mean ± SEM) (Fig. 6B)*.* In both wt-AAV2 and MAAP mutant construct-transfected cells the capsids accumulated into the nucleus (Fig. 6C)*.* The comparison of nuclear and cytoplasmic intensities indicated that transfection with wt-AAV2 and MAAP mutants resulted in relatively similar levels of nuclear localisation of viral capsids.

Taken together, our analyses demonstrated the dual association of MAAP in the nuclear periphery in close proximity to or occasionally in the nuclear envelope membranes, and with the plasma membrane. As a result of the MAAP mutations, only relatively slight changes in the intracellular localisation of capsid proteins or capsids were observed. However, the deletions of MAAP altered virion production, suggesting that MAAP might have a potential regulatory role either in the nuclear export or cellular egress of progeny viral capsids through the plasma membrane.

### Mutated *MAAPs* produce unstable and truncated peptides

**Figure 7:**
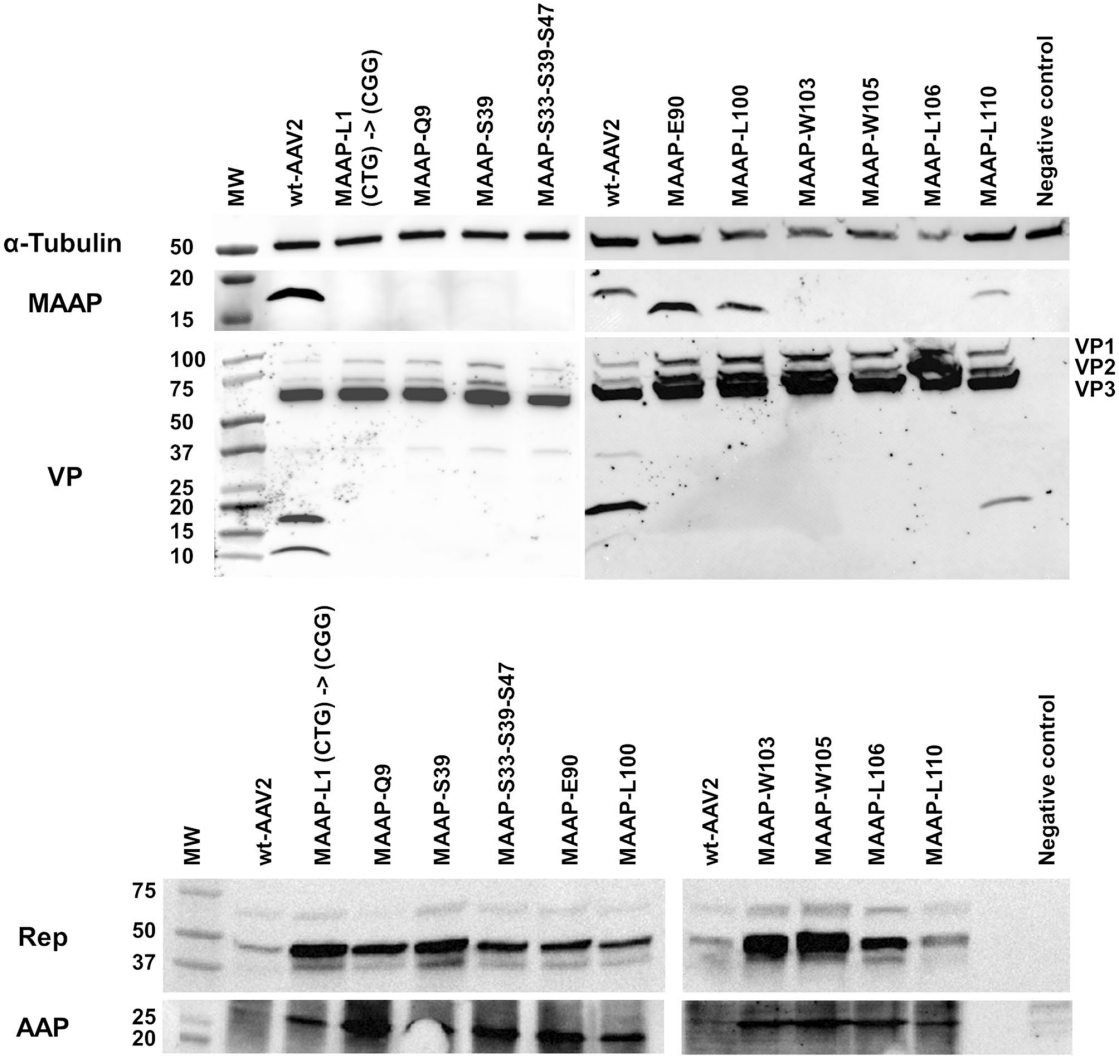
MAAP variants and their effect on AAV proteins. To study the effects of MAAP variants on capsid proteins, 293 T cells were co-transfected with wt-AAV2 or MAAP variant plasmids and Ad helper plasmid. From cell extracts harvested at 24 hpt, we detected α-Tubulin, MAAP, VPs, Rep and AAP by immunoblotting.

Using the MAAP specific antibody, we were able to detect wt-MAAP from the wt-AAV2 and Ad helper plasmid co-transfected cells. Besides wt-MAAP, the antibody allowed detection of MAAP mutants with a stop codon introduced at the 3’- end of the ORF. Interestingly, while some of the mutants (MAAP-E90, MAAP-L100, MAAP-L110) were produced as stable truncated forms visible in immunoblot, others (MAAP-W103, MAAP-W105, MAAP-L106) were undetectable (Fig. 7). The fate of the further c-terminally truncated MAAP variants remain unknown as the antibody was raised against aa 79-98 of MAAP (Fig. 1, underlined). Some stable truncated variants may retain residual wt-MAAP activity.

### Truncation and inactivation of MAAP results in higher Rep and AAP protein expression levels

We measured the effect of MAAP mutations on the Rep and AAP protein expression (Fig. 7). All MAAP variants induced a slight increase in large Rep protein amount, possibly Rep68, and a much larger induction of small Rep detection, possibly Rep52. The smallest increase was observed for the MAAP-L110 variant encoding almost the full length MAAP. A global increase of AAP expression was observed for all MAAP variants, with the smallest expression increase seen for MAAP-L110.

### MAAP induces VP degradation

To study the impact of MAAP on capsid integrity, an antibody recognising the C-terminal end of all VPs was used to characterise the generated wt- as well as MAAP-mutated viruses. In wt-AAV2 and Ad helper-transfected 293 T cells, VP degradation products began to form as early as at 21 hpt^29^ (Fig. 3). The sizes of these VP specific peptides were about 32 kDa (Fig. 3 dash), 18 kDa (Fig. 3 asterisk), and 12 kDa (Fig. 3 hash). Interestingly, every MAAP variant except for MAAP-L110 allowed virus production without a sign of VP degradation (Fig. 7). In accordance, we detected an improved level of VP1 and VP2 over VP3. The MAAP-L110 still displayed the specific 18 kDa VP degradation product but with lower intensity than wt-AAV2. This lower degree of VP degradation could result from the lower production of MAAP-L110 in cells evident by immunoblotting and microscopy experiments or the impaired MAAP function associated with the C-terminal end, MAAP2BR3 domain (Fig. 1). When recombinant MAAP was produced in complement to the AAV variant MAAP-S33-S39-S47, proteolytic activity towards the capsid proteins was restored (S3 Fig).

**Figure 8:**
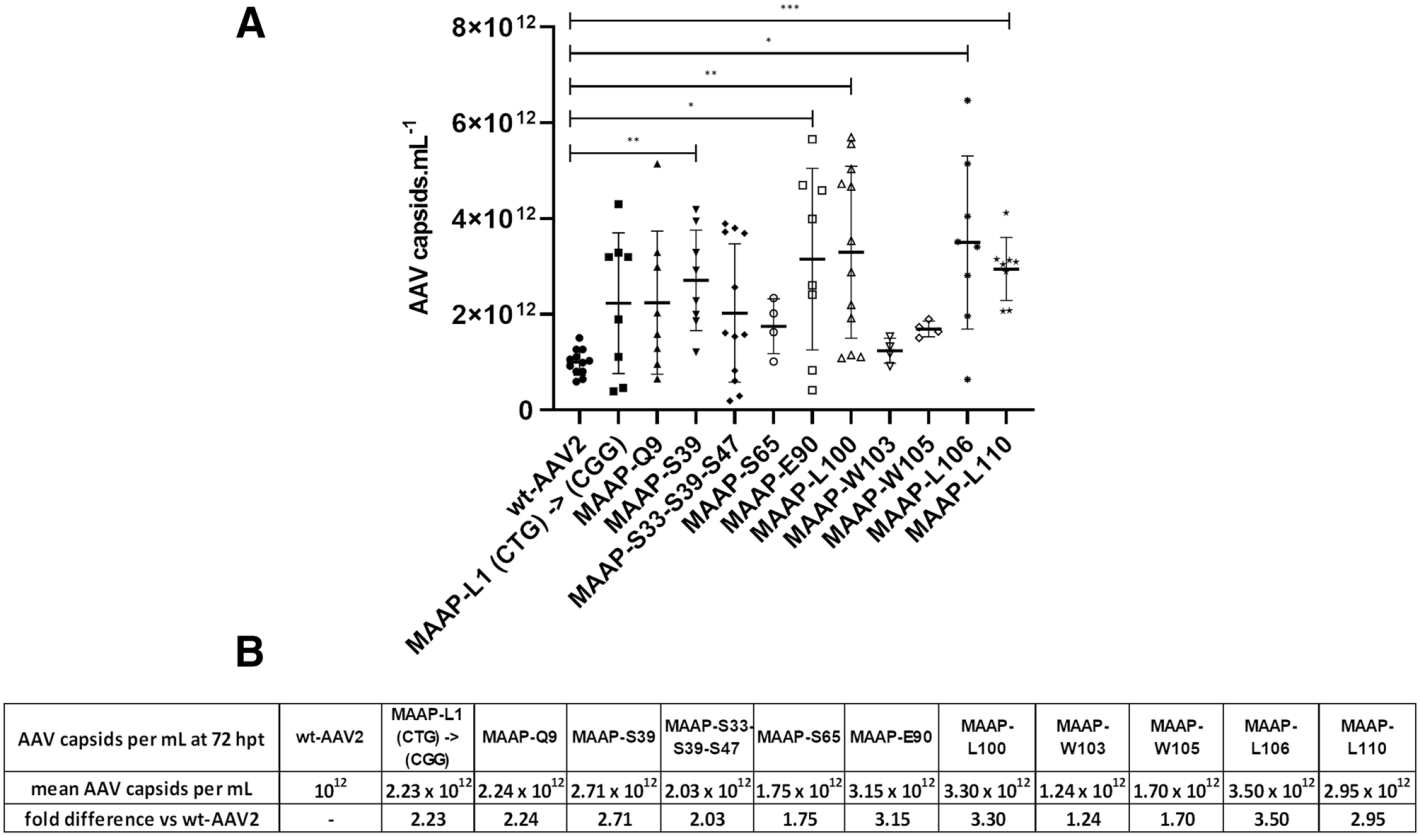
MAAP effects on capsid levels. Plasmids with wt-AAV2 or AAV2 encoding MAAP variants were co-transfected with Ad helper plasmid. From cell extracts harvested 72 hpt, we performed AAV2 capsids ELISA (**A**), expressed as AAV capsids per mL with mean and the standard deviation (SD). Statistical significance between wt-AAV2 and AAV2 encoding MAAP mutants was evaluated using ANOVA followed by Dunnett’s multiple comparison test. Table (**B**) indicates the average AAV per mL capsid titer measured for each virus, along with the fold difference compared to wt-AAV2.

As suggested during the microscopy experiments, and as observed on Western blot, both studied at 24 hpt, when MAAP expression is impaired or when C-terminal truncated forms of MAAP are produced, VPs levels increase. For MAAP variants at 72 hpt, ELISA analysis showed up to 3.5-fold increase in assembled capsids (Fig. 8). This may be a consequence of both reduced capsid degradation and higher capsid assembly that were observed. The reason for a diminished variance associated to wt-MAAP is unknown.

### Impact of MAAP inactivation and truncation on AAV genome packaging

**Figure 9:**
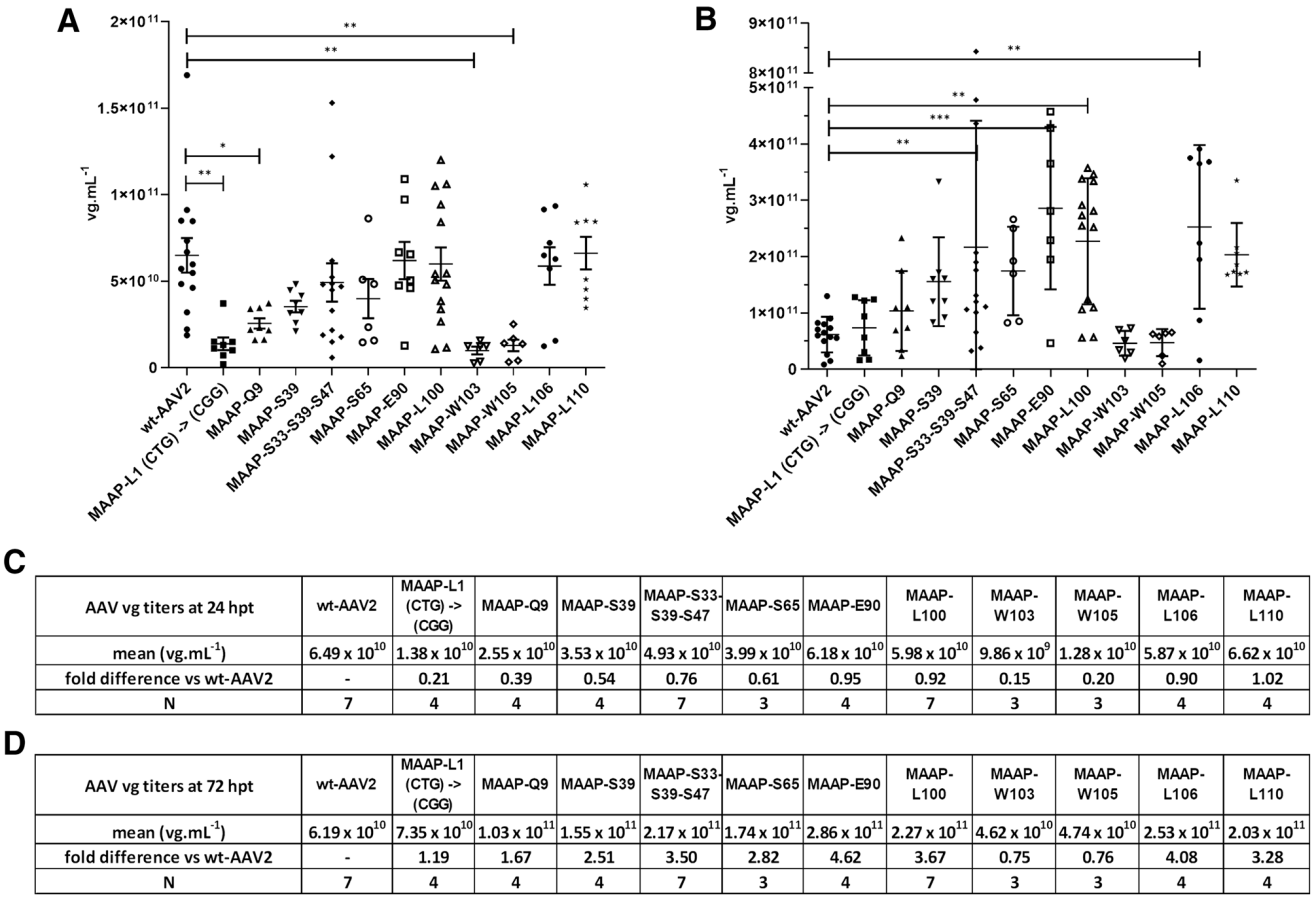
Effect of MAAP inactivation on AAV2 vg titers. wt-AAV2 or AAV2 MAAP variants and Ad helper plasmids were co-transfected in 293 T cells. At 24 hpt (**A**) and 72 hpt (**B**) AAV vg titers were quantified, with results expressed as vg mL^−1^ with mean and SD. Statistical significance between wt-AAV2 and AAV2 MAAP variants was evaluated using ANOVA followed by Dunnett’s multiple comparison test. The table represents the average vg mL^−1^ titer measured for each virus, along with the fold difference compared to wt-AAV2 at 24 hpt (**C**) and 72 hpt (**D**). Experiments were performed independently between 3 and 7 times (indicated as N), each time with two replicate samples.

Interestingly, compared to wt-AAV encoded MAAP, all variants except MAAP-L110 displayed either a slight or a more drastic reduction in viral genome titers (vg mL^−1^) at 24 hpt. The most prominent drop of 85% was measured for MAAP-W103 (Fig. 9A,C). Among the MAAP variants resulting in the highest titers (close to wt-AAV2) were the ones for which a C-terminally truncated version could be detected in immunoblotting, excluding MAAP-L106. Inactivation of the MAAP start codon or the mutants with early ORF stop codons displayed markedly reduced titers over wt-AAV2. At 24 hpt, wt-MAAP thus seems to provide a replicative advantage to wt-AAV2 over the MAAP-variants. At 72 hpt, however, compared to wt-AAV2, only MAAP-W103 and MAAP-W105 showed reduced titers (0.75- and 0.76-fold, respectively) (Fig. 9B,D); for all other MAAP variants, the titers were improved, up to 4.6-fold observed for MAAP-E90.

**Figure 10:**
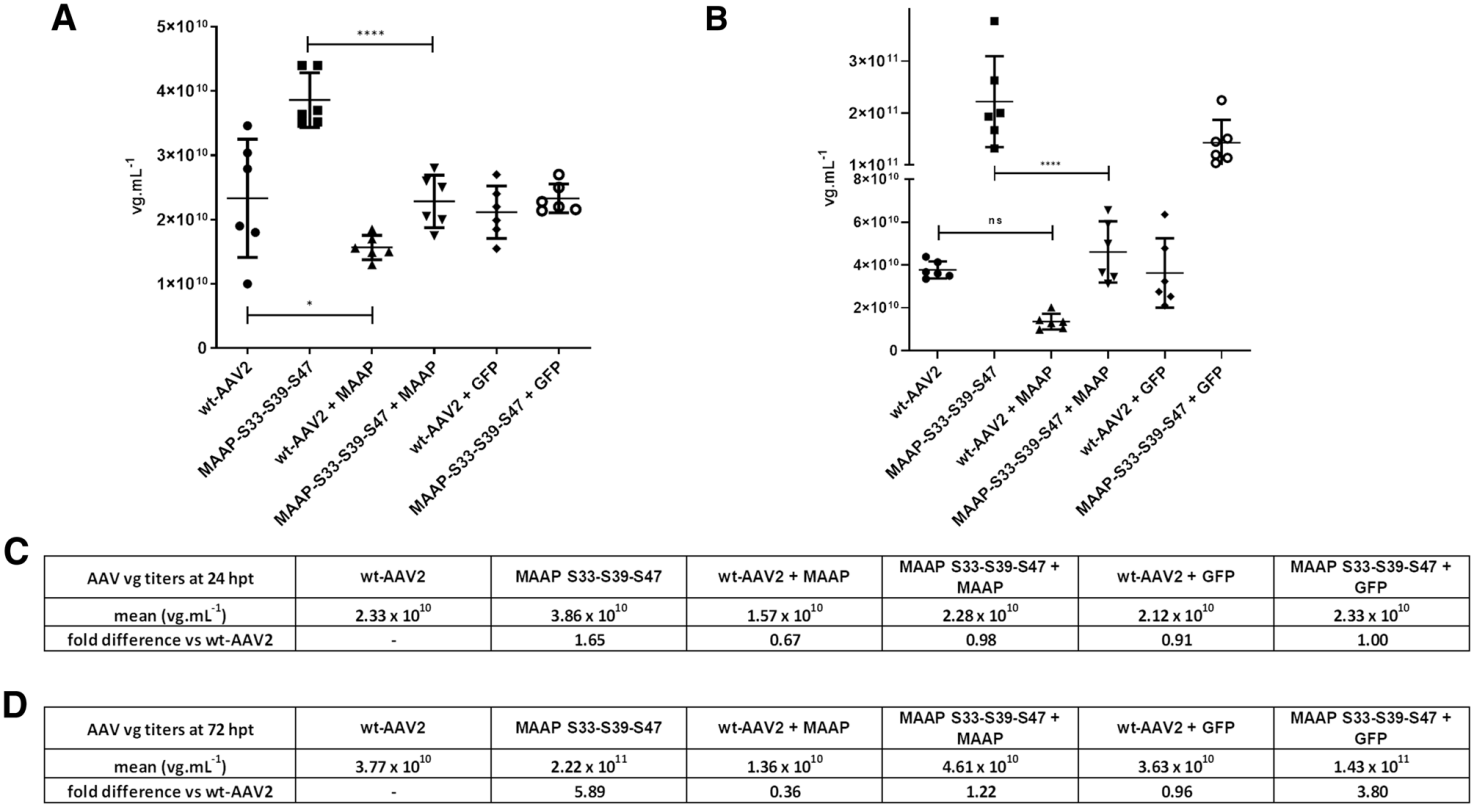
MAAP trans-complementation. We performed wt-AAV2 and MAAP-S33-S39-S47 variant production with or without the addition of recombinant MAAP. Vg titers (vg mL^−1^) were measured from cell extract samples harvested 24 hpt (**A**) and 72 hpt (**B**). Graphics show individual samples with mean and SD. Statistical significance was evaluated for recombinant MAAP addition using ANOVA followed by Dunnett’s multiple comparison test. Tables (**C**) and (**D**) represent the average titer (vg mL^−1^) at 24 hpt and 72 hpt, with fold difference to wt-AAV2. The samples from left to right are: (1) wt-AAV2. (2) MAAP-S33-S39-S47. (3) wt-AAV2 trans-complemented with MAAP expressing plasmid. (4) MAAP-S33-S39-S47 trans-complemented with MAAP expressing plasmid. (5) wt-AAV2 trans-complemented with a GFP plasmid of similar size to the recombinant MAAP plasmid. (6) MAAP-S33-S39-S47 complemented with a GFP plasmid of similar size to the recombinant MAAP plasmid.

We wanted to know how recombinant MAAP overproduction affects wt-AAV2 and MAAP-S33-S39-S47 variant titers. Compared to plain wt-AAV2 production, the overproduction caused viral titers to decrease by 33% at 24 and 64% at 72 hpt (Fig. 10A–D). A GFP control plasmid of similar size as MAAP plasmid reduced the viral titers only by 9% at 24 hpt and 4% at 72 hpt. When MAAP-S33-S39-S47 was applied to virus production, the titers increased 1.65-fold at 24 hpt and 5.89-fold at 72 hpt. MAAP overexpression in combination with MAAP-S33-S39-S47, however, resulted in similar vg titers as those with wt-AAV2 at 24 hpt, and only 1.22-fold increase at 72 hpt. Co-transfection with the GFP control plasmid gave titers equal to those of the wt-AAV2 reference at 24 hpt, and 3.80-fold higher at 72 hpt. The overproduction of MAAP suggested that compared to other viral or host proteins, potentially AAP and/or VPs, it is required in correct stoichiometric levels in infection.

### MAAP effects on contaminating DNA packaging into AAV

**Figure 11:**
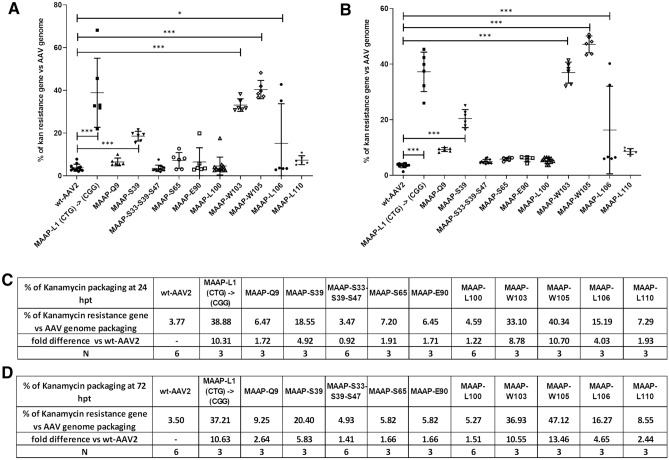
Effect of MAAP inactivation on kanamycin resistance gene packaging. wt-AAV2 or AAV2 MAAP variants and Ad helper plasmids were co-transfected in 293 T cells. At 24 hpt (**A**) and 72 hpt (**B**), AAV vg titers were quantified. In parallel, the kanamycin resistance gene carried by the AAV2 genome and the Ad helper plasmids was quantified. We present the ratio of kanamycin resistance gene packaging relative to the AAV2 genome packaging, expressed as percentage. Individual samples are represented, along with the mean and the standard deviation. Statistical significance between the wt-AAV2 and AAV encoding MAAP mutants was evaluated using ANOVA followed by Dunnett’s multiple comparison test. Tables (**C**) and (**D**) show the average percentage of kanamycin resistance gene packaging relative to AAV2 genome packaging, measured for each virus at 24 hpt and 72 hpt, and the fold difference compared to wt-AAV2. Experiments were performed independently between 3 and 6 times (indicated as N), each time with two samples.

We also studied the effect of MAAP on the packaging of contaminating DNA originating from the producer plasmids into the AAV capsid. The kanamycin resistance gene is present in both the Ad helper and wt-AAV2 encoding plasmids. Compared to genome packaging, we measured antibiotic contamination in wt-AAV2 viruses of 3.77% at 24 and 3.50% 72 hpt (Fig. 11A–D). At 24 and 72 hpt for all MAAPs bearing mutation close to the ORF 5’-end, excluding MAAP-S33-S39-S47, and for the 3’-end mutants for which no MAAP protein expression was detected, kanamycin resistance gene packaging increased up to tenfold compared to wt-AAV2 (Fig. 11A–D). The highest contamination level was observed for MAAP-W105 at 72 hpt at 13.46-fold over wt-AAV2, kanamycin resistance gene accounting for 47.12% compared to AAV genome. MAAP-S33-S39-S47 and variants that produced a stable C-terminal truncated form of MAAP (MAAP-E90, MAAP-L100 and MAAP-L110) showed similar or only slightly higher kanamycin gene packaging than wt-AAV2 both at 24 and 72 hpt. When MAAP was complemented (overexpressed) in wt-AAV2 or MAAP-S33-S39-S47 production, kanamycin resistance gene packaging was increased compared to the wt-AAV2 control at both time points (S4 Fig).

To study whether the antibiotic resistance gene packaging originated preferentially from the Ad helper or wt-AAV2 encoding plasmid, we measured the packaging of adenovirus E4 gene. When MAAP-L1 CTG start codon was modified to CGG, or when MAAP-W103 and MAAP-W105 was used, at 24 hpt, E4 gene packaging increased over fourfold (S5 Fig). At 72 hpt, the difference between wt-AAV2 and the MAAP variants ranged from a 0.32 to 2.2-fold, but without a statistical significance (S5 Fig). Altogether, the results suggest that in the absence of MAAP production, most of the contaminating DNA originates from the wt-AAV2 genome plasmid. The AAV ITRs consist of a palindromic hairpin (HP) structure and a 20-nucleotide stretch, the D-sequence, not involved in the HP formation^30^. For an AAV genome inserted in a plasmid (double-stranded DNA), the HP is sought to arrange as an Holliday-structure^30,31^, and due to the symmetrical nature of the HP, only the D-sequence allows the selective recognition of the AAV genome over the plasmid backbone. Our results suggest that MAAP could be involved in ITR-mediated genome packaging through a selective recognition of the D sequence.

### MAAP affects AAV genome packaging in the capsid

**Figure 12:**
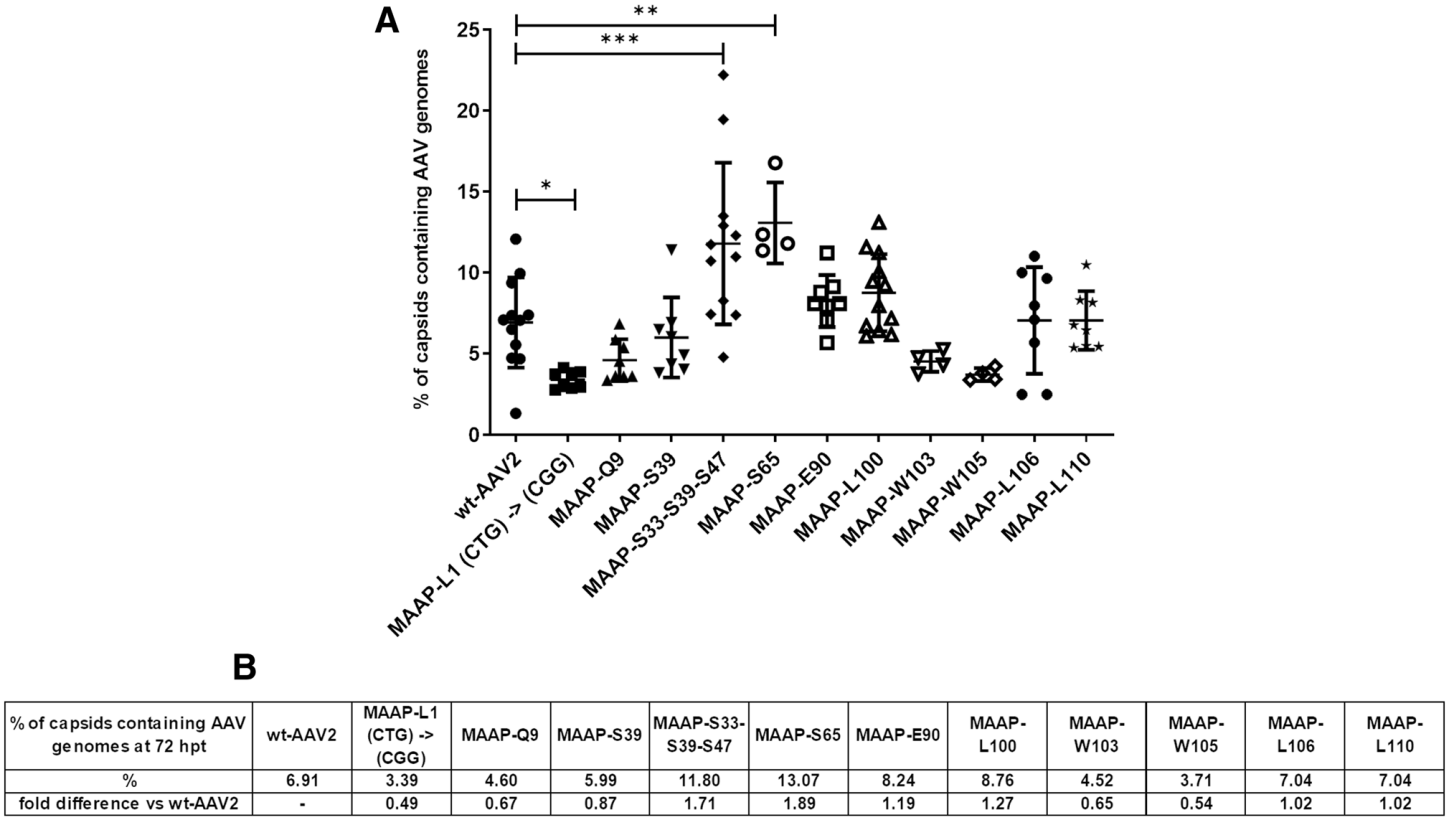
Effect of MAAP inactivation on genome packaging. wt-AAV2 or AAV2 MAAP variants and Ad helper plasmids were co-transfected in 293 T cells. At 72 hpt (**A**) AAV vg titers were quantified. In parallel, we quantified the total number of AAV capsids from the same samples by ELISA. We present the ratio of capsid containing AAV2 genome versus total capsids, expressed as percentage. Samples are represented, with mean and SD. Statistical significance between wt-AAV2 and AAV encoding MAAP variants was evaluated using ANOVA followed by Dunnett’s multiple comparison test. Table (**B**) shows the average percentage of capsid containing AAV2 genomes and the fold difference compared to wt-AAV2.

To find out if MAAP is involved in genome packaging, we measured the proportion of capsids filled with the genome compared to the total number of capsid. Of the wt-AAV2 capsids, 6.91% contained the genome (Fig. 12). A significant decline in the genome content was observed when MAAP translation was halted by start codon modification (MAAP-L1) or when stop codon was introduced into MAAP ORF resulting in no detectable MAAP production (MAAP-Q9, MAAP-S39, MAAP-W103 and MAAP-W105). Interestingly, these mutants also encapsidated higher levels of kanamycin resistance gene, as described above. As opposite, especially MAAP-S33-S39-S47 and MAAP-S65 gave higher quantities of genome-containing viruses.

### MAAP accelerates AAV replication in co-infection with adenovirus

**Figure 13:**
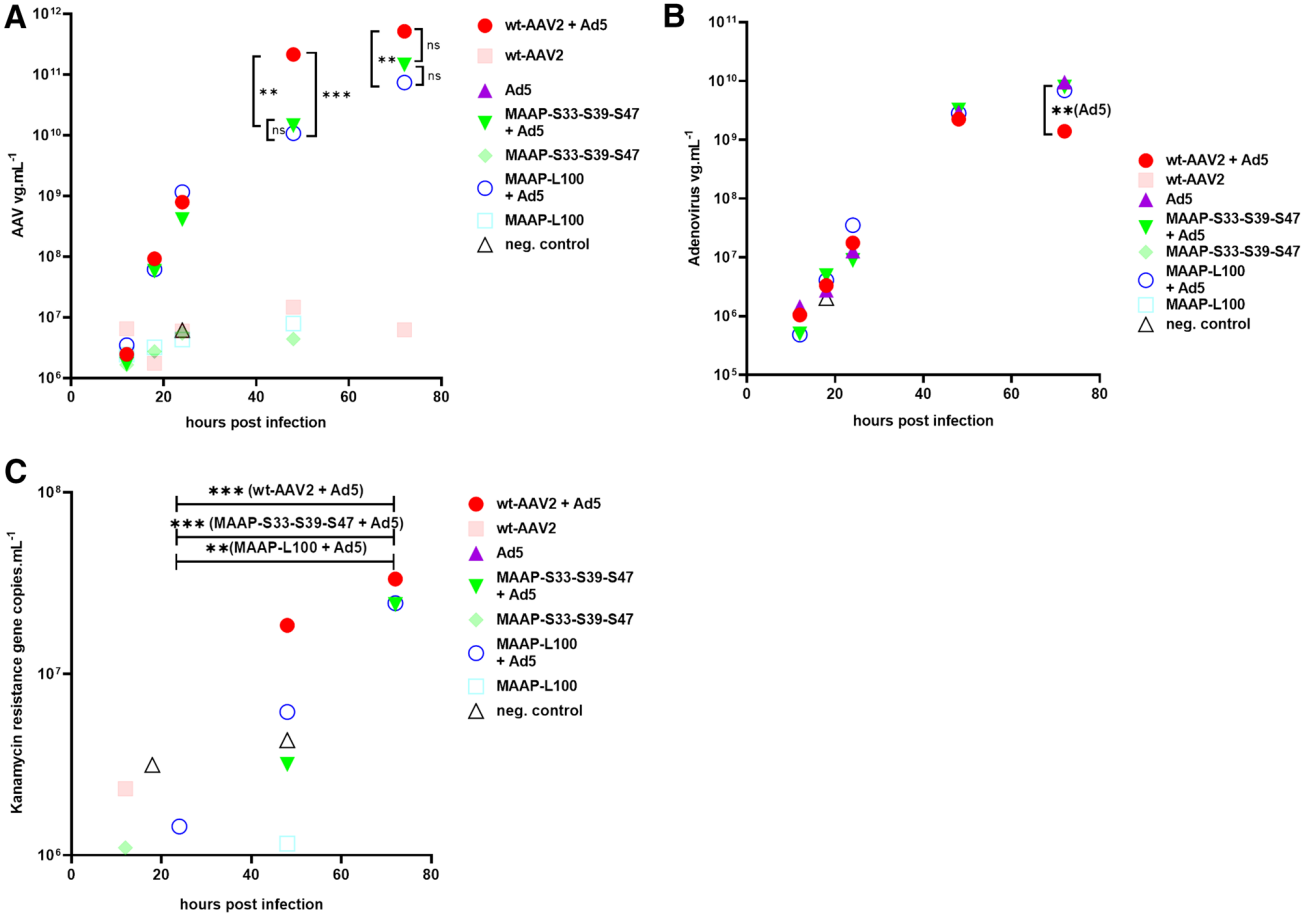
MAAP inactivation and its effect, in the context of AAV2 and Adenovirus 5 co-infection. We generated wt-AAV2, AAV2-MAAP-S33-S39-S47 and AAV2-MAAP-L100 viruses using plasmid transfection of 293 T cells. The harvested viruses were used to infect 293 T cells at an MOI of 500 with or without Adenovirus 5 added at an MOI of 50. Samples were harvest at 12, 18, 24, 48, 72 hpi and AAV vg titers (**A**), adenovirus vg titers (**B**), and kanamycin resistance gene titers (**C**) were quantified. Medians of the titers are represented. Statistical significance between samples was evaluated using Kruskal–Wallis test followed by Dunn’s multiple comparison test.

To find out how MAAP affects AAV2 and adenovirus co-infection, we produced wt-AAV2, MAAP-S33-S39-S47 and MAAP-L100 viruses and used them to co-infect 293 T cells with serotype 5 adenovirus. All viruses grew exponentially for over 72 h (Fig. 13A). We did not observe differences between wt-AAV2 and the variants until 24 h post infection (hpi). However, at 48 hpi, the MAAP modified viruses yielded significantly lower vg titers than wt-AAV2. At 72 hpi, the titers of AAV2-MAAP-S33-S39-S47 and AAV2-MAAP-L100 were still lower than that of wt-AAV2. Thus, MAAP seems to act as an accelerating factor for wt-AAV2. A consequence of more rapid replication of the wt-AAV2 is a reduction of the adenovirus replication (Fig. 13B), in accordance with^32^. The levels of AAV capsids containing the AAV genome did not significantly differ between the wt-AAV2 and the MAAP variant viruses (S6 Fig). We observed an increase in the proportion of capsids containing AAV genomes both for wt-AAV2 and MAAP variants during the kinetic. These levels are higher at 72 hpi than what we observed during the production of the AAV viruses using plasmid transfection.

We also studied the kanamycin resistance gene contamination (after AAV production) during the co-infection (Fig. 13C). Interestingly when wt-AAV2 or the MAAP mutant viruses were used with adenovirus, between 24 and 72 hpi, kanamycin resistance gene copies significantly increased. The amplification was not detected in the AAV2 infected cells or when only adenovirus was used to infect the 293 T cells. The contamination potentially originated from the AAV2 ITR-plasmid backbone (encoding the kanamycin resistance gene) packaged during virus production, which was amplified and newly packaged into AAV2 capsids during the co-infection. This raises a potential safety concern for AAV-mediated gene therapy^33^. A case of a treated patient being exposed to wt-AAV and helper virus co-infection could lead to amplification and packaging of the contaminant DNA (potentially associated to ITR sequences) into progeny AAV viruses.

## Discussion

Since the discovery of AAV2 in 1965 as a contaminant of adenovirus preparation, AAVs have been extensively studied, leading them to become one of the most used vectors today in gene therapy^34,35^. However, a deeper understanding of AAV biology is necessary for resolving existing obstacles in AAV-mediated gene delivery. Gene therapies typically require very large doses of AAV and improvements are needed to boost virus production yields. Similarly, gene therapy patients are more likely to mount an immunological response from virus preparations which contain large fractions of empty capsids or contaminating DNA^36,37^.

In this work, we studied the AAV2-encoded protein MAAP for which the role in AAV biology is still mainly undefined. Our results extend the recent findings^19^ that this novel viral protein is the result of a frameshifted gene in the VP1 region and that translation begins from the first CTG (L1) codon in the MAAP ORF. We have further demonstrated that replacing the CTG (L1) codon to CGG (R1) prevents MAAP production. Likewise, introducing a TAG stop codon in the place of CAG (Q9) prevented MAAP translation that otherwise could have begun from the sub-optimal, downstream start codons, AGG (R13) and ACG (T14). Contrary to MAAP-GFP fusion-protein-based findings^19^, we were not able to visualise any secondary MAAP translation by MAAP specific antibody when the CTG (L1) start codon was inactivated.

Here we report a set of MAAP mutations which are highly beneficial in AAV production, both in terms of virus quantity and quality. We further report the natural replicative advantage that MAAP confers over Ad-helper-virus in the context of natural infection.

Our confocal microscopy studies and image analyses showed that MAAP associates with the cell membrane in transfected cells. Here we have shown MAAP in close proximity to nuclear membrane, as well as in other intracellular locations. In silico protein-structure predictions have conjectured that MAAP contains a membrane-binding motif—a hydrophobic amphipathic α-helix at the C-terminal position, aa 95-116^27^. Our microscopy provides additional evidence of this purported membrane-association. The amphipathic α-helix is known to function as an anchor of peripheral proteins on membranes^38–40^, as well to sense membrane curvature. The amphipathic α-helix also has a role in promoting the membrane curvature necessary for membrane fusion^41–43^. This curvature of intracellular membranes is a well-modelled, essential component of enveloped RNA virus budding^44^. The influenza M2 protein, for instance, uses an amphipathic helix for membrane deformation and scission during viral budding from the host cell^42,45,46^. Alternatively, in brome mosaic virus (BMV) protein 1a, the amphipathic α-helix is essential for association with the ER membrane and for perinuclear ER localisation^47^. Hepatitis C virus also depends on amphipathic α-helix of the NS5A and NS4B proteins during assembly of the HCV replication complex. The membrane anchor function of α-helix is required for the association of these proteins with ER membranes and the nuclear membrane^48–51^. Among non-enveloped DNA viruses, adenoviruses are notable in containing a N-terminal amphipathic α-helix in the pVI protein that induces membrane fragmentation and rupture required for endosomal escape of viral particles during cell entry^52–54^. Adenoviruses also encode another membrane binding protein known as the adenovirus death protein (E3A-11.6 K protein, ADP) which contains a single membrane-spanning domain. We postulate that the MAAP bears the greatest similarity to ADP due to their shared size and profound positive charges at physiological pH (see below). The action of ADP is to localise at the ER and Golgi apparatus where the protein goes through N- and O-glycosylation, after which it is cleaved and transported to the inner nuclear membrane, where it facilitates host cell death via inducing membrane rupture^55^.

We supply that the predicted amphipathic α-helix of MAAP arises near to BR clusters at the C-terminal end of the MAAP. These BR clusters function as a nuclear localisation signal (NLS) by presenting high homology to the AAP BR clusters known to provide this function^22^. Our own (almost) complete deletion of the MAAPBR3 did not provide evidence of impaired nuclear localisation, so it remains that the MAAP2BR1 and MAAP2BR2 domains may still have allowed for membrane association. We suspect that super-resolution imaging might reveal some subtle subcellular differences between the MAAP variants, especially considering that the absence of MAAP2BR3 already led to marked changes in AAV production.

The observation that MAAP is associated with the plasma membrane as well as with intracellular membranes, such as nuclear envelope and perinuclear ER, is also consistent with notions that cytosolic leaflets of biological membranes are enriched in negatively charged lipids. MAAP is characterised by an unusually high positive charge at cellular pH having an isoelectric point (pI) of 12 and hydrophobic and positive residues of amphipathic α-helices^27^, which enable hydrophobic and electrostatic interactions to membrane. Despite the knowledge that MAAP interacts with intracellular membranes, many questions remain regarding the detailed molecular basis of MAAP functions.

MAAP’s association with the plasma membrane suggests that it may also play a role in the modification of membrane structure and permissiveness, and thereby could influence virus exit from the host cell. Cellular egress of non-enveloped viruses such as AAV2 is generally thought to occur as a virus burst after apoptotic disintegration of the host cell. Studies of autonomous parvoviruses have suggested that virus-induced DNA damage responses promote apoptosis. For example, minute virus of mice (MVM), rat parvovirus H-1 and human B19 viruses induce caspase dependent apoptosis in infected cells^56–59^. Previous research has shown that the AAV2 Rep78 protein induces caspase activation and apoptosis^60^. In cells co-infected with AAV-2 and adenovirus, apoptosis is detected even in the absence of adenovirus-induced cytolysis^61^. However, the specific mechanisms by which AAV-2 progeny viruses are released from the host cell, and whether this release happens through the plasma membrane or from apoptotic vesicles released from cells at the final stage of infection, remains to be determined. Our findings demonstrate that the deletion or mutation of MAAP decreases the progeny virus yield in natural co-infection set-up. This suggests that accumulation of MAAP to the plasma membrane may play a role in enhancing capsid egress. This is consistent with the notion that MAAP seems to affect extracellular secretion of AAV virions^62^. Some of our preliminary findings in transfected point in this direction as well, but we cannot yet rule out that the decrease in production of infectious progeny is due to the alternative roles of MAAP during pre-apoptotic steps of infection e.g. capsid assembly and transport. The possible roles of MAAP in facilitation of production and transport of progeny capsids warrants additional studies in infected cells.

We note here that MAAP had a strong effect on capsid (proteins) integrity. The lack of only the last ten amino acids at the MAAP C-terminus boosted VP and capsid levels and reduced capsid degradation while the total deletion of MAAP2BR3 fully prevented the degradation of AAV2 capsid. Proteasome inhibition plays a role during AAV infection and the addition of protease inhibitor can prevent capsid antigen presentation^63^, as well enhance viral transduction^64^. Furthermore, AAV capsid has been shown to be able to auto-cleave in acidic conditions^65^, and AAV capsids are subjected to proteasome-involved post-translational modifications (PTMs) during wt-AAV production, including ubiquitination^66^. These PTMs could potentially be used as signals to initiate host cell defence or to down-regulate new capsids via ubiquitination and subsequent proteasomal degradation^67^. In addition to a possible impact of MAAP on the cellular degradation processes, the AAV stabilising effect of MAAP could be achieved by protecting the capsids from entering into subcellular localisations where the degradation process takes place. However, we did not observe an effect of MAAP on capsid egress from the nucleus within 24 hpt. There remain significant open questions regarding viral egress which warrant further investigation. Notably, it is unknown whether egress is halted in later time points, MAAP can stealth capsids, or whether secretion into the medium is simply reduced.

Our data further suggests that MAAP is involved with the packaging of AAV genome. Some MAAP variants lost the capacity to selectively package the AAV genome over ITR encoding plasmid DNA. One mutant in particular seemed to retain ITR specificity although we had truncated its MAAP to only the N-terminal portion. This putative genome-refining function is once again supported by MAAP’s small size and strong positive charge which makes it capable of binding to negatively charged DNA. These properties could also give MAAP the capacity to act as a transcriptional regulator, which would enable the detection and modulation of capsid production levels as well as AAP and Rep protein expression levels, possibly in concert with Reps.

In conclusion, our results provide new insights into the intracellular interactions and function of MAAP, a recently described AAV protein. Our results show that MAAP is targeted to the cytoplasmic membranes and nuclear envelope, and thereby has a potential role in the virus-membrane interactions essential for progression of infection. Consistent with this model we showed that MAAP modifications substantially improve AAV capsid yield and quality. This suggests that MAAP may give AAV an important replicative advantage over the helper virus in the natural co-infection context but is not essential for AAV replication.

## Material and methods

### Virus preparations

AAV2 Virus production were prepared as follows. 293 T cells (European Collection of Cell Cultures 293 T Number: 12022001) were grown in Dulbecco's modified Eagle medium (DMEM, Gibco 11965084) supplemented with 10% fetal bovine serum (FBS, Thermo Fisher 10091-148), supplemented with 2 mM L-glutamine (Gibco, 25030-024), and penicillin–streptomycin (Gibco 15070-063). Polyethylenimine (PEI) transfections of AAV2 plasmid and adenovirus helper plasmid (1:1 ratio, total 350 ng/cm^2^) were performed on 293 T cells in T25 flasks (60,000 cells/cm^2^). The cell density reached 70–90% confluence at the time of transfection. The PEI Pro (Polyplus Transfection, ref# 115-100)/DNA weight ratio was maintained at 1:1 in serum-free DMEM medium. Virus was harvested 24 h and 72 h after transfection. For viral genome titer determination and AAV2 capsid ELISA samples, virus was harvested using Triton-X-100 buffer (0.5% Triton-X-100 (Sigma-Aldrich, ref# X100-1L) and 2 mM MgCl2 (Merck, ref# E13980) in 1 × PBS (Gibco, ref# 18912-014)) and Denarase (50 U/ml, c-Lecta, ref# 20804-5 M). Lysis buffer was added to the media and cells were incubated for 2 h at 37 ˚C before cell lysate was collected. Crude lysates were used in the experiments without further purification.

For samples processed for Western blot, virus was harvested as follows. Cells were detached using TrypLE Select (Gibco, ref# 12563-011) and suspended in 1 × PBS (Gibco, ref# 14190-094). Cells were pelleted by centrifugation (500 g, 5 min). Cell pellet was washed with 1 × PBS and centrifugation was repeated. Cells were resuspended in radioimmunoprecipitation assay (RIPA, Thermo Scientific, ref# 89901) buffer containing Proteinase Inhibitor Cocktail (cOmplete, Roche, ref# 1169749800). Samples were incubated on ice for 20 min and centrifuged at 20 000 g for 15 min. Supernatant was collected.

Human adenovirus 5 (ATCC VR-1516) amplification was performed in 293 T cells. The cells were grown in T175 flasks (68 500 cells/cm^2^) in DMEM (Gibco 11965084) supplemented with 10% fetal bovine serum (FBS, Thermo Fisher 10091-148), 2 mM L-glutamine (Gibco, 25030-024), and penicillin–streptomycin (Gibco 15070-063). From glycerol working stock, adenovirus 5 was added to the flasks with multiplicity of infection (MOI) of 40. Flasks were monitored every consecutive day until detaching cells were observed (62 h post infection). Cells were detached completely using cell scrapers and the suspension was spun at 1 100 g for 10 min at room temperature (RT). Pellet was resuspended in 1 × PBS (Gibco, ref# 14190-094), frozen with liquid nitrogen, fully thawed and vortexed. The freeze/thaw process was repeated 3 times. Cell lysate was spun at 4 °C for 20 min at 2 000 g. Cell debris from the supernatant were filtered out using a 0.45 µM syringe filter. Glycerol (Merck, EMPROVE, 1.04093.2500) was added to the filtrate to a final volumetric concentration of 10% and stored at − 80 °C.

### Plasmids

Plasmids were obtained by gene synthesis from Genewiz. A complete list is provided in Supplementary Table S1.

### AAV2 and adenovirus infections

AAV2 and adenovirus (co)-infections were performed with AAV2 virus samples harvested at 24 hpt and stored at 4 °C prior to use, while the adenovirus 5 samples were harvested at 62 hpi and frozen prior to use (both prepared as described in section “virus preparation”). For the experiment, AAV2 and adenovirus 5 samples (crude lysates) were used at MOIs of 500 and 50, respectively, for (co)-infections on 293 T cells. Cells were grown in supplemented DMEM media on Poly-l-lysine (Merck, ref# P4707)-coated 12-well plates (37 000 cells/cm^2^). Media was changed 6 hpi (1 ml/well). Viruses were harvested 12, 18, 24, 48, and 72 hpi.

### Quantification of AAV genomes, producer plasmid sequences, and adenovirus 5 genomes by droplet digital PCR

To obtain droplet digital PCR (ddPCR) AAV vg titers, crude preparations of virus were DNaseI (0.01 U/µl, Invitrogen, ref# 18047-019) and Proteinase K (0.1 µg/µl, Roche, ref# 03115879001) treated, and viral titers were obtained by ddPCR amplification (QX200, Bio-Rad) with primers (Rep2-FWD; Rep2-REV) and probe (Rep2-PRB) detecting AAV replicase region (Supplementary Table S2). To assess levels of packaged DNA originating from the AAV plasmid backbone and adenovirus helper plasmid backbone, ddPCR was performed using primers (Kan-FWD; Kan-REV) and probe (Kan-PRB) for the kanamycin resistance gene. The adenovirus E4 (Ad5-E4) region set of primers (Ad5-E4-FWD; Ad5-E4-REV) and probe (Ad5-E4-PRB) was used to quantify the adenovirus helper plasmid and adenovirus 5. All primers and probes were ordered from Integrated DNA Technologies. For mastermix generation, primers (900 nM) and probe (250 nM) were diluted in 2 × ddPCR Supermix for Probes (no dUTP, Bio-Rad, ref# 1863025) and nuclease free water (Thermo Scientific, ref#R0582).

### ELISA

To determine the ratios of capsids containing AAV2 genomes from total AAV2 capsids, A20 capsid ELISA was first performed on 500–10,000 serial dilutions of the virus preparation using the AAV2 titration ELISA kit (Progen, ref # PRATV) according to the manufacturer’s instructions. Ratios were then calculated by dividing AAV vg titers by capsid titers.

### Western blotting

Samples were heat denatured using 2-mercaptoethanol (10%, Sigma-Aldrich) in Laemmli sample buffer (Bio-Rad, #1610747). Maximum volume was run on Mini-Protean TGX gels (4–10%, Bio-Rad). Proteins were transferred to 0.2 µm PVDF membrane (Trans-Blot Turbo Transfer Pack, Bio-Rad) and stained with selected primary antibody overnight at 4 °C. Proteins were detected with horseradish peroxydase (HRP) conjugated secondary antibody, and visualised using ChemiDoc (Bio-Rad). The AAV Replicase proteins were detected using the primary antibody “anti-AAV2 Replicase mouse monoclonal, 303.9” (Progen, #61069) diluted to 1:250 and the “Goat anti Mouse IgG (H + L)-HRP Conjugate” (Bio-Rad, #170-6516) secondary antibody diluted to 1:3000. The AAV Capsid proteins were detected using the primary antibody “anti-AAV VP1/VP2/VP3 mouse monoclonal, B1” (Progen, #61058) and the “Goat anti Mouse IgG (H + L)-HRP Conjugate” (Bio-Rad, #170-6516) secondary antibody diluted to 1:3000. The Polyclonal MAAP antiserum was obtained from the immunization of a rabbit with peptide KKIRLLGATSDEQSSRRKRG (MAAP aa 79-98), conjugated to a carrier before immunization and affinity purified (MAAP GAL-KKI, Davids Biotechnology). For MAAP detection in Western blot experiments, the antiserum was diluted to 1 µg mL^−1^, and the “Goat Anti-Rabbit IgG (H + L)-HRP Conjugate” (Bio-Rad, #170-6515) secondary antibody diluted to 1:3000. The polyclonal AAP antiserum was obtained from the immunization of a Guinea pig with peptide RSTSSRTSSARRIKDASRR, conjugated to a carrier before immunization and affinity purified (Davids Biotechnologie). For AAP detection in Western blot experiments, the antiserum was diluted to 3 µg mL^−1^, and the “Anti-guinea pig IgG (H + L)-HRP conjugate” (Sigma, #SAB3700379) secondary antibody diluted to 1:1000. The α-tubulin was detected using α-tubulin HRP conjugated mouse monoclonal IgG (Santa Cruz Biotechnology, #sc-32293 HRP) diluted to 1:1000. Protein molecular mass weight (MW) marker was “Precision Plus Protein Dual Color Standards” from Bio-Rad (ref #161-0374).

### Statistical analysis

The statistical analyses were performed using GraphPad software (Prism). The significance values shown above the bars on the figures are denoted as **** (*p* < 0.0001), *** (*p* < 0.001), ** (*p* < 0.01), * (*p* < 0.05) or ns (not significant).

### Flow cytometry analysis

Flow cytometry samples were prepared starting from transfected 293 T cells as described in the section ‘Virus Preparations’. At 24 hpt, cells were detached using TrypLE Select (Gibco, ref# 12563-011). 1 × PBS (Gibco, ref# 14190-094) with 4% Paraformaldehyde (Sigma-Aldrich, ref# 158127-500G) was added in volumetric ratio of 1:1 to fix the cells. Flow cytometry samples were then run on CytoFLEX S Flow cytometer (Beckman Coulter Inc.) with acquisition settings FSC = 151, SSC = 209 and FITC = 10. Data analysis was performed using CytExpert software version 2.4.0.28 (Beckman Coulter, Inc.).

### Confocal microscopy

For immunolabelling studies, 293 T cells, cultured on glass coverslips, were co-transfected with AAVwt-AAV, MAAP variants MAAP-S33-S39-S47, MAAP-W105, MAAP-L106, MAAP-L110, and the adenovirus helper plasmid, or transfected with the MAAP expression plasmid. Cells were fixed at 24 hpt with 4% PFA and permeabilised with 0.1% Triton X-100 in PBS supplemented with 0.5% BSA. MAAP was detected with a specific polyclonal antibody (Ab) (MAAP GAL-KKI, Davids Biotechnologie), viral capsid proteins with a monoclonal antibody (MAb) against VP1, VP2 and VP3 (B1, Progen, #61084), and intact viral capsids with a MAb (A20, Progen, #61055). A20 recognises both empty and full (DNA containing) capsids. The primary antibodies were followed by goat anti-mouse or anti-rabbit Alexa-448 or Alexa-546 conjugated secondary Abs (Thermo Fisher Scientific). The chromatin was stained in the embedding stage either with 4′-6-diamidino-2-phenylindole (DAPI) or with NucBlue (Thermo Fisher Scientific, Massachusetts, USA).

The cells were imaged using Leica TCS SP8 FALCON laser scanning confocal microscope (Leica microsystems, Mannheim, Germany) applying HC PL APO CS2 63x/1.40 oil immersion objective (NA 1.4). Alexa 488 and Alexa 546 were excited with 499 nm and 557 nm wavelengths, respectively, of pulsed white light laser (80 MHz). Emission detection range was 505–561 nm for Alexa 488 and 562–701 nm for Alexa 546. DAPI and NucBlue were excited with a 405 nm diode laser and emission between 410 and 504 nm were detected. Image stack size was 512 × 512 with a pixel size of 60 nm/pixel in the x- and y-directions, and step size of 120 nm in the z-direction.

Fluorescent label intensities were calculated by separating 3D confocal microscopy stacks of cells into the cytoplasm and nucleus using Otsu's automatic thresholding algorithm^68^ for DAPI signal and then summing the pixel intensities inside and outside the segmented nucleus. The mean total intensity in the nucleus and cytoplasm and their standard errors were calculated over every imaged cell in the sample. The MAAP intensity as a function of the distance from the nuclear envelope was calculated by segmenting the nuclei using Otsu's method^68^ and by calculating for every pixel the shortest Euclidean distance to the nuclear border. The distance values were sorted into 2 pixel (120 nm) wide bins, and the mean pixel intensity was calculated for every bin. Surface renderings of MAAP and DAPI were created with Leica TCS SP8 FALCON.

## Supplementary Information

Supplementary Information.

Supplementary Video.
