# Supplementary material for: Functional roles of the membrane-associated AAV protein MAAP
Source: Sci Rep. 2021 Nov 4;11:21698. doi: 10.1038/s41598-021-01220-7 (PMC8568889; doi:10.1038/s41598-021-01220-7)
Supplement: Supplementary file 1 — Supplementary Information. [file 41598_2021_1220_MOESM1_ESM.docx]

Functional roles of the membrane-associated AAV protein MAAP

Lionel Galibert, Amira Hyvönen, Reetta A.E. Eriksson, Salla Mattola, Vesa Aho, Sami Salminen, Justin D. Albers, Sanna K. Peltola, Saija Weman, Tiina Nieminen, Seppo Ylä-Herttuala, Hanna P. Lesch, Maija Vihinen-Ranta, Kari J. Airenne

**Supplementary information**

**S1 Fig. MAAP-GFP expressed from the AAV2 genome.**

GFP fused to MAAP was expressed in the context of the AAV2 genome plasmid, with or without Ad helper plasmid, in 293T cells. GFP expression was measured by FACS and expressed as median of fluorescence intensity (MFI). From left to right, AAV2 encoding: (1) wt-MAAP-GFP. (2) MAAP-GFP - L1 (CTG) start codon modified to R (CGG). (3) MAAP-GFP-Q9. (4) MAAP-GFP-S33. (5) MAAP-GFP-S39. (6) MAAP-GFP-S47. (7) MAAP-GFP-S33-S39-S47. (8) MAAP-S33-S39-S47. (9) wt-MAAP-GFP co-transfected with Ad helper plasmid. (10) MAAP-GFP - L1 (CTG) start codon modified to R (CGG) co-transfected with Ad helper plasmid. (11) MAAP-GFP-Q9 co-transfected with Ad helper plasmid. (12) MAAP-GFP-S33 co-transfected with Ad helper plasmid. (13) MAAP-GFP-S39 co-transfected with Ad helper plasmid. (14) MAAP-GFP-S47 co-transfected with Ad helper plasmid. (15) MAAP-GFP-S33-S39-S47 co-transfected with Ad helper plasmid. (16) MAAP-S33-S39-S47 co-transfected with Ad helper plasmid. (17) non-transfected 293T cells. Statistical significance was evaluated for recombinant MAAP addition using ANOVA.

**S2 movie. 3D surface rendering of wt-AAV2 MAAP.**

3D surface reconstruction of confocal z-stacks showing the MAAP protein distribution in green and the nucleus labelled by DAPI in blue. Surface rendering was created with Leica TCS SP8 FALCON 3D viewer.


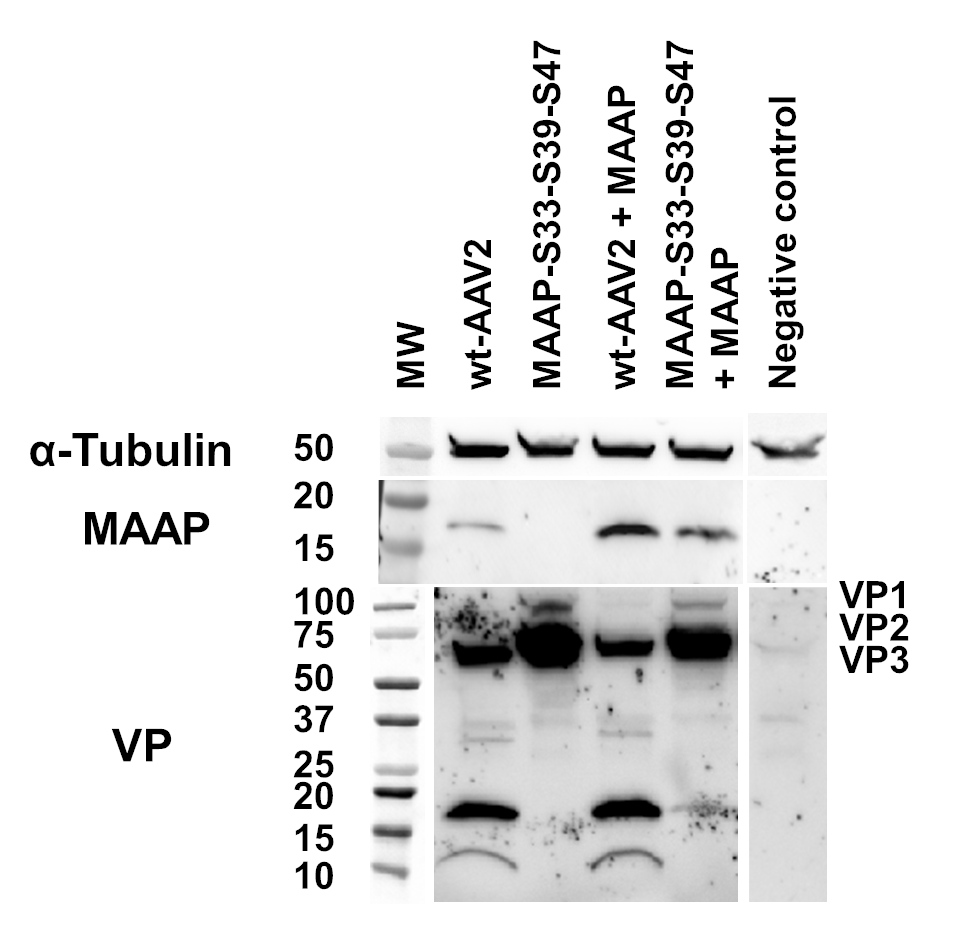


**S3 Fig. MAAP trans-complementation.**

The effect of MAAP trans-complementation during the production of wt-AAV2 and MAAP-S33-S39-S47 variant was studied. From cell extracts harvested 24 hpt, we performed the immunostaining of α-Tubulin (top panel), MAAP (middle panel), and VPs (lower panel). The samples from left to right are: (1) MW. (2) wt-AAV2. (3) MAAP-S33-S39-S47. (4) wt-AAV2 trans-complemented with recombinant MAAP plasmid. (5) MAAP-S33-S39-S47 trans-complemented with recombinant MAAP plasmid. (6) non-transfected 293T cells.


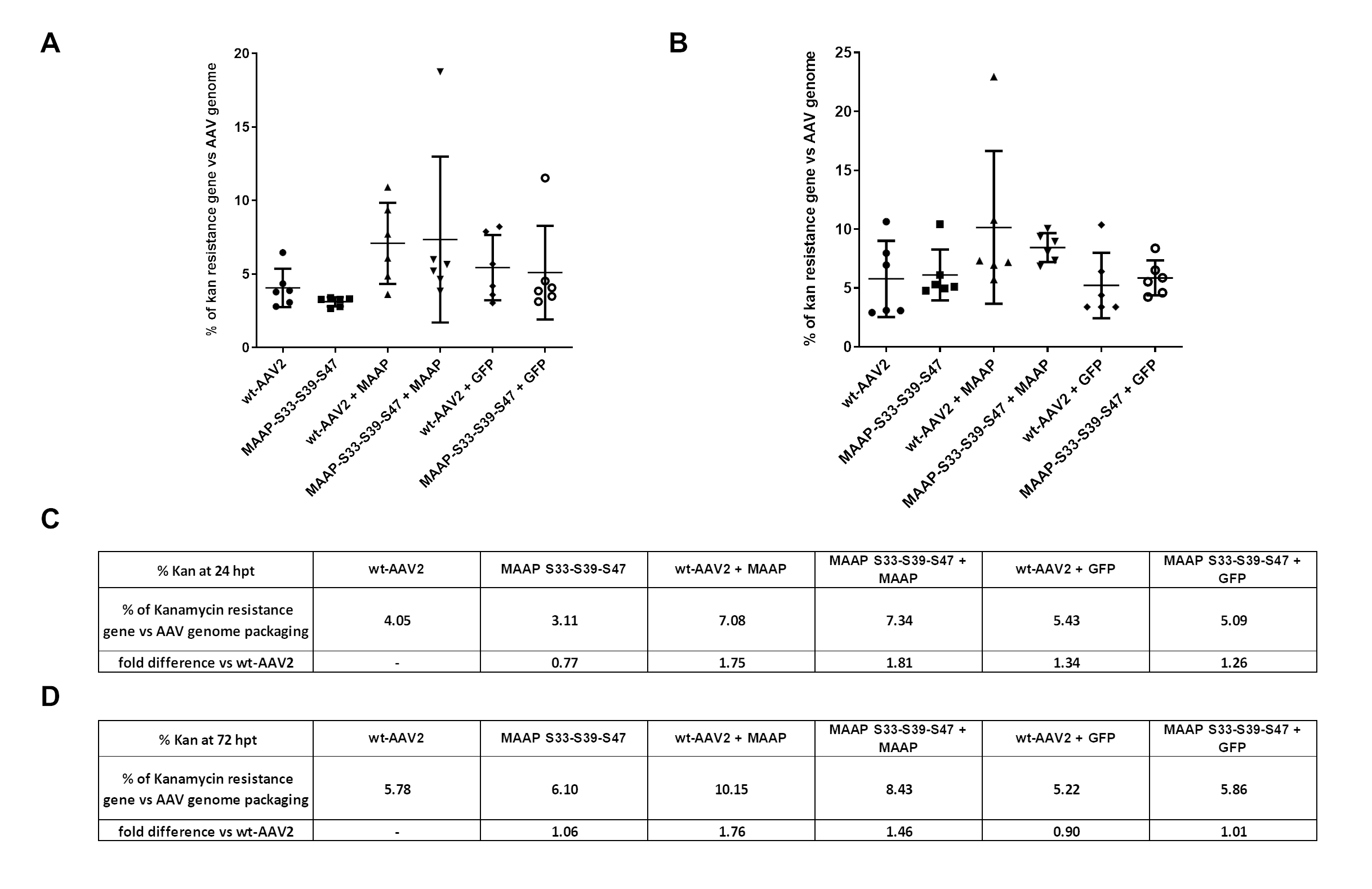


**S4 Fig. Effect of MAAP trans-complementation on kanamycin resistance gene packaging.**

We performed wt-AAV2 and MAAP-S33-S39-S47 variant production with or without the addition of recombinant MAAP and quantified vg titers (vg.mL^-1^) from cell extract samples harvested 24 hpt (**A**) and 72hpt (**B**). In parallel, the kanamycin resistance gene, originally carried by the AAV2 and Ad helper plasmids, was quantified. We present the ratio of kanamycin resistance gene packaging relative to the AAV2 genome packaging, expressed as percentage.

Graphics show individual samples with mean and SD. Statistical significance was evaluated for recombinant MAAP addition using ANOVA followed by Dunnett’s multiple comparison test. Tables **(C)** and **(D)** show the average percentage of kanamycin resistance gene packaging relative to AAV2 genome packaging at 24 hpt and 72 hpt, with fold difference to wt-AAV2. The samples from left to right are: (1) wt-AAV2. (2) MAAP-S33-S39-S47. (3) wt-AAV2 trans-complemented with MAAP expressing plasmid. (4) MAAP-S33-S39-S47 trans-complemented with MAAP expressing plasmid. (5) wt-AAV2 trans-complemented with a GFP plasmid of similar size to the recombinant MAAP plasmid. (6) MAAP-S33-S39-S47 complemented with a GFP plasmid of similar size to the recombinant MAAP plasmid.


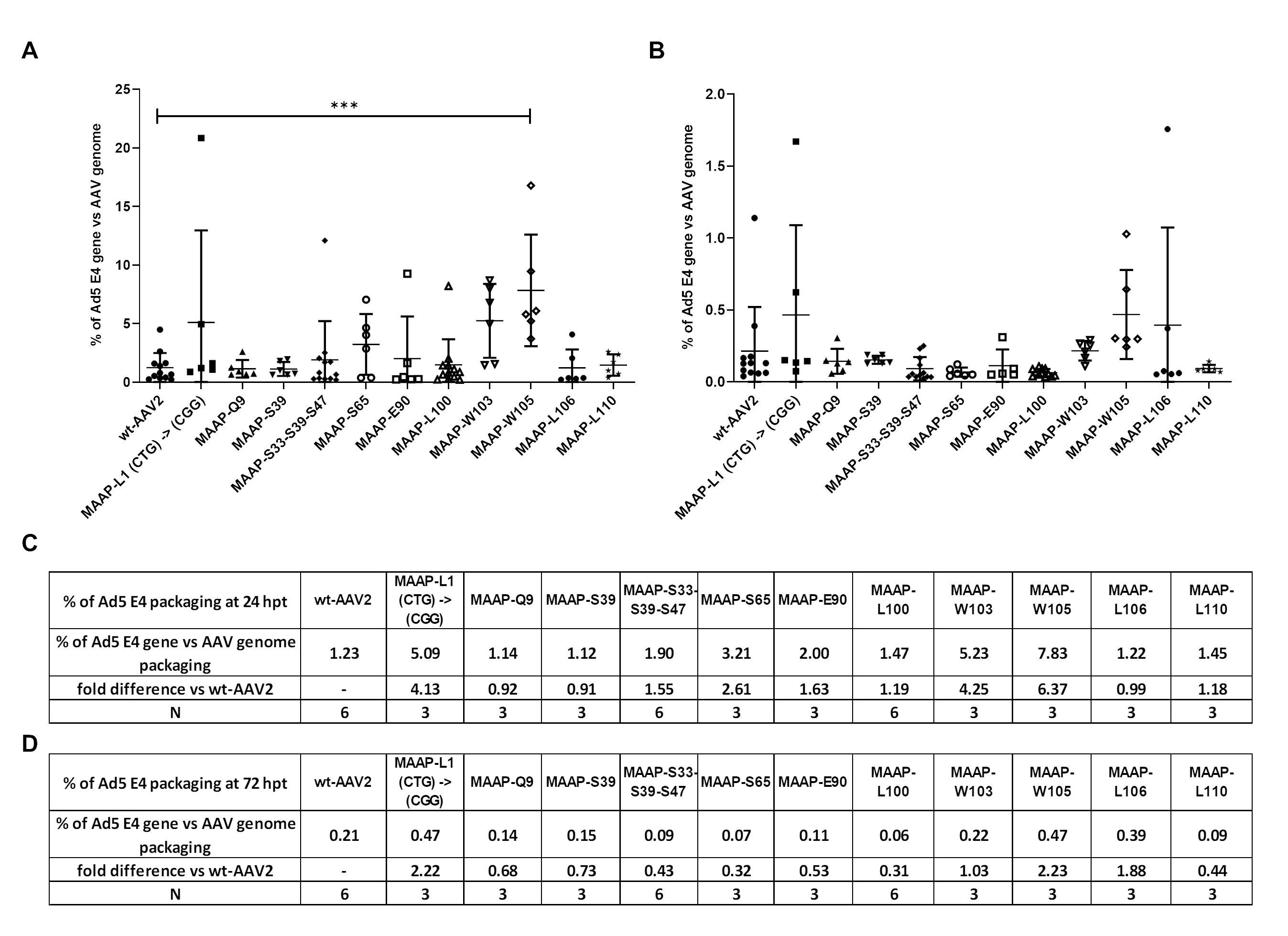


**S5 Fig. Effect of MAAP inactivation on Adenovirus E4 gene packaging.**

wt-AAV2 or AAV2 MAAP variants and Ad helper plasmids were co-transfected in 293T cells. At 24 hpt **(A)** and 72 hpt **(B)**, AAV vg titers were quantified. In parallel, the adenovirus E4 gene carried by the Ad helper plasmid, was quantified. We present the ratio of adenovirus E4 gene packaging relative to the AAV2 genome packaging, expressed as percentage. Individual samples are represented, along with mean and standard deviation. Statistical significance between the wt-AAV2 and AAV encoding MAAP mutants was evaluated using ANOVA followed by Dunnett’s multiple comparison test. Tables **(C)** and **(D)** show the average percentage of adenovirus E4 gene packaging relative to AAV2 genome packaging, measured for each virus at 24 hpt and 72 hpt, and the fold difference compared to wt-AAV2. Experiments were performed independently between 3 and 6 times (indicated as N), each time with two samples.


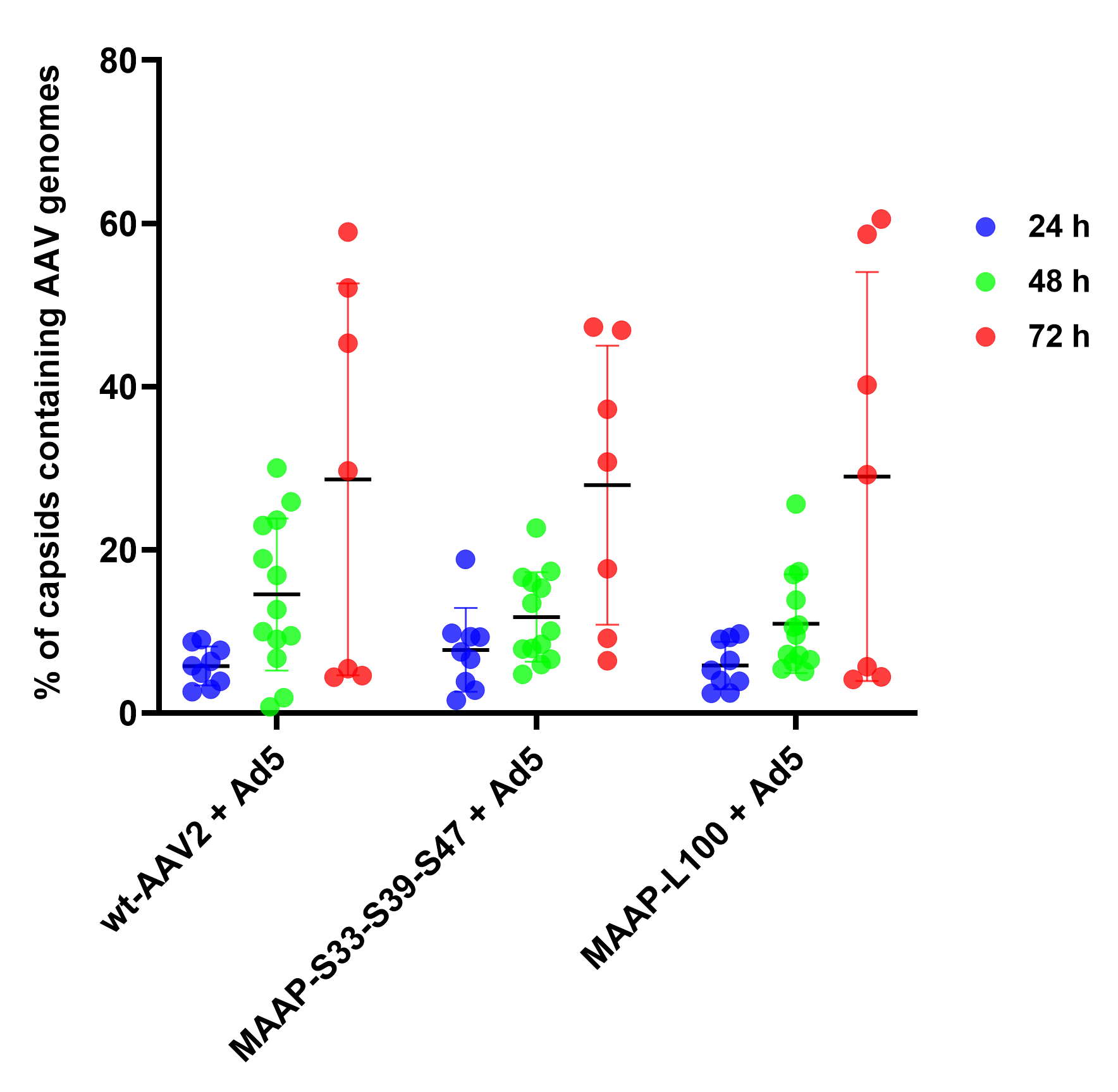


**S6 Fig. Effect of MAAP inactivation on genome packaging, in the context of AAV2 and Adenovirus 5 co-infection.**

We generated wt-AAV2, AAV2-MAAP-S33-S39-S47 and AAV2-MAAP-L100 viruses using plasmid transfection of 293T cells. The harvested AAV viruses were used to infect 293T cells at an MOI of 500, with or without adding Adenovirus 5 at an MOI of 50. Samples were harvest at 12, 18, 24, 48, and 72 hpi and AAV vg titers were quantified. In parallel, from the same samples, we quantified the total number of AAV capsids by ELISA. We present the ratio of capsid containing AAV2 genome versus total capsids, expressed as percentage. Samples are represented, with mean and SD.

**Supplementary Table S1. Plasmids used in this study**

| **plasmid number** | **plasmid name ^a^** | **referred as** | **MAAP original codon and amino acid** | **MAAP mutation** | **VP1 original codon and aa** | **VP1 aa following MAAP mutation** | **plasmid size (bp)** | **Purpose of the plasmid** |
| --- | --- | --- | --- | --- | --- | --- | --- | --- |
| p0059 | p0059 - pDEST-eGFP |  |  |  |  |  | 3030 | Stuffer plasmid of similar size recombinant MAAP plasmids (p0280 - p0326) |
| p0088 | p0088 - pHelper-Ad5-Lio |  |  |  |  |  | 14540 | Adenovirus helper plasmid used for wt-AAV2 production. Encodes VAI, VAII, E2, E4 regions of Adenovirus 5. |
| p0108 | p0108 - pGRG25-AAV2WT |  | WT sequence | - | WT sequence | - | 17225 | Reference for wt-AAV2 production (Savy et al., 2018) ^b^ |
| p0188 | p0188 - AAV2WT-DS Q16 -> stop | MAAP-Q9 | Gln 9 (CAG) | stop 9 (TAG) | Ala 35 (GCA) | Val 35 (GTA) | 6952 | Study of MAAP start codon in wt-AAV2 plasmid context |
| p0189 | p0189 - AAV2WT-DS S40-S46-S54 -> stops | MAAP-S33-S39-S47 | Ser 33 (TCG) - Ser 39 (TCA) -Ser 47 (TCG) | Stop 33 (TAG) - Stop 39 (TGA) - Stop 47 (TAG) | Leu 59 (CTC) - Val 65 (GTC) - Leu 73 (CTC) | Leu 59 (CTA) - Val 65 (GTA) - Leu 73 (CTA) | 6952 | Study of MAAP in wt-AAV2 plasmid context |
| p0190 | p0190 - AAV2WT-DS S40 -> stop | MAAP-S33 | Ser 33 (TCG) | Stop 33 (TAG) | Leu 59 (CTC) | Leu 59 (CTA) | 6952 | Study of MAAP in wt-AAV2 plasmid context |
| p0191 | p0191 - AAV2WT-DS S46 -> stop | MAAP-S46 | Ser 39 (TCA) | Stop 39 (TGA) | Val 65 (GTC) | Val 65 (GTA) | 6952 | Study of MAAP in wt-AAV2 plasmid context |
| p0192 | p0192 - AAV2WT-DS S54 -> stop | MAAP-S47 | Ser 47 (TCG) | Stop 47 (TAG) | Leu 73 (CTC) | Leu 73 (CTA) | 6952 | Study of MAAP in wt-AAV2 plasmid context |
| p0193 | p0193 - AAV2WT-DS S72 -> stop | MAAP-S65 | Ser 65 (TCA) | Stop 65 (TGA) | Leu 91 (CTC) | Leu 91 (CTC) | 6952 | Study of MAAP in wt-AAV2 plasmid context |
| p0194 | p0194 - AAV2WT-DS E97 -> stop | MAAP-E90 | Glu 90 (GAG) | Stop 90 (TAG) | Arg 116 (CGA) | Leu 116 (CTA) | 6952 | Study of MAAP in wt-AAV2 plasmid context |
| p0195 | p0195 - AAV2WT-DS L107 -> stop | MAAP-L100 | Leu 100 (TTG) | Stop 100 (TAG) | Leu 126 (CTT) | Leu 126 (CTA) | 6952 | Study of MAAP in wt-AAV2 plasmid context |
| p0196 | p0196 - AAV2WT-DS W110 -> stop | MAAP-W103 | Trp 103 (TGG) | Stop 103 (TAG) | Leu 129 (CTG) | Leu 129 (CTA) | 6952 | Study of MAAP in wt-AAV2 plasmid context |
| p0197 | p0197 - AAV2WT-DS W112 -> stop | MAAP-W105 | Trp 105 (TGG) | Stop 105 (TAG) | Leu 131 (CTG) | Leu 131 (CTA) | 6952 | Study of MAAP in wt-AAV2 plasmid context |
| p0198 | p0198 - AAV2WT-DS L113 -> stop | MAAP-L106 | Leu 106 (TTG) | Stop 106 (TAG) | Val 132 (GTT) | Val 132 (GTA) | 6952 | Study of MAAP in wt-AAV2 plasmid context |
| p0199 | p0199 - AAV2WT-DS L117 -> stop | MAAP-L110 | Leu 110 (TTA) | Stop 110 (TGA) | Val 136 (GTT) | Val 136 (GTG) | 6952 | Study of MAAP in wt-AAV2 plasmid context |
| p0200 | p0200 - AAV2WT-DS-GFP | MAAP-GFP | WT sequence | WT sequence | insertion of GFP fused to MAAP takes place after VP1-P145 |  | 7666 | Study of MAAP start codon - MAAP-GFP fusion in wt-AAV2 plasmid context |
| p0201 | p0201 - AAV2WT-DS-GFP Q16 -> stop | MAAP-GFP-Q9 | Gln 9 (CAG) | Stop 9 (TAG) | Ala 35 (GCA) | Val 35 (GTA) | 7666 | Study of MAAP start codon - MAAP-GFP fusion in wt-AAV2 plasmid context |
| p0202 | p0202 - AAV2WT-DS-GFP S40 -> stop | MAAP-GFP-S33 | Ser 33 (TCG) | Stop 33 (TAG) | Leu 59 (CTC) | Leu 59 (CTA) | 7666 | Study of MAAP start codon - MAAP-GFP fusion in wt-AAV2 plasmid context |
| p0203 | p0203 - AAV2WT-DS-GFP S46 -> stop | MAAP-GFP-S39 | Ser 39 (TCA) | Stop 39 (TGA) | Val 65 (GTC) | Val 65 (GTA) | 7666 | Study of MAAP start codon - MAAP-GFP fusion in wt-AAV2 plasmid context |
| p0204 | p0204 - AAV2WT-DS-GFP S54 -> stop | MAAP-GFP-S47 | Ser 47 (TCG) | Stop 47 (TAG) | Leu 73 (CTC) | Leu 73 (CTA) | 7666 | Study of MAAP start codon - MAAP-GFP fusion in wt-AAV2 plasmid context |
| p0205 | p0205 - AAV2WT-DS-GFP S40-46-54 -> stops | MAAP-GFP-S33-S39-S47 | Ser 33 (TCG) - Ser 39 (TCA) -Ser 47 (TCG) | Stop 33 (TAG) - Stop 39 (TGA) - Stop 47 (TAG) | Leu 59 (CTC) - Val 65 (GTC) - Leu 73(CTC) | Leu 59 (CTA) - Val 65 (GTA) - Leu 73 (CTA) | 7666 | Study of MAAP start codon - MAAP-GFP fusion in wt-AAV2 plasmid context |
| p0206 | p0206 - AAV2WT-DS-GFP start1 Leu8 -> Arg | MAAP-L1 (CTG)-> (CGG) | Leu 1 (CTG) | Arg 1 (CGG) | Pro 27 (CCT) | Pro 27 (CCG) | 7666 | Study of MAAP start codon - MAAP-GFP fusion in wt-AAV2 plasmid context |
| p0230 | p0230-pUC-K-AAV2WT-Reverse |  | WT sequence | - | WT sequence | - | 6952 | Reference for wt-AAV2 production |
| p0273 | p0273 - Ad5-Lio-v7 |  |  |  |  |  | 13123 | Adenovirus helper plasmid used for wt-AAV2 production. Encodes VAI, VAII, E2, E4 regions of Adenovirus 5. |
| p0280 | p0280 - Death Star | pMAAP |  |  |  |  | 3284 | Expression of MAAP driven by ATG codon, under CMV enhancer - CMV promoter - SV40 intron promoter sequence |
| p0283 | p0283 - Death Star-GFP | MAAP-GFP |  |  |  |  | 3998 | Expression of MAAP-GFP driven by ATG codon, under CMV enhancer - CMV promoter - SV40 intron promoter sequence |
| p0326 | p0326 - Death Star start 2 | MAAP-Start2 |  |  |  |  | 3248 | Expression of MAAP initiated at Start 2 (R13) modified to ATG codon, under CMV enhancer - CMV promoter - SV40 intron promoter sequence |

^a^ When we initiated this study, MAAP wasn’t yet discovered and published. We initially named the MAAP protein Death Star (DS), reflected in the name of the plasmids. The ORF initiation codon was also unknown and numbering of the DS amino acids in the plasmid names started according to the first amino acid following the previous stop codon of the MAAP ORF.

^b^ Savy, A. et al. Genetics instability of wtAAV2 genome and AAV promoter activities in the Baculovirus/Sf9 cells system. PLoS One 13, e0199866 (2018).

**Supplementary Table S2. The primers and probes used in the study:**

| **Primer/probe name** | **Sequence and dye** |
| --- | --- |
| Rep2-PRB | 5’-6-FAM /CCCGTGTCA/ZEN/GAATCTCAACCCGTT/IABkFQ |
| Rep2-FWD | 5’-CTTCACTCACGGACAGAAAGA |
| Rep2-REV | 5’-CTGGCACCTTTCCCATGATA |
| Ad5-E4-PRB | 5’-6-FAM /ACCCAGCCA/ZEN/ACCTACACATTCGTT/IABkFQ |
| Ad5-E4-FWD | 5’-CATCCACCACCGCAGAATAA |
| Ad5-E4-REV | 5’-ACATGGTTCTTCCAGCTCTTC |
| Kan-PRB | 5’-6-FAM /TCGCACCTG/ZEN/ATTGCCCGACATTAT/IABkFQ |
| Kan-FWD | 5’-ATCGGGCTTCCCATACAATC |
| Kan-REV | 5’-GCTCTAGGCCGCGATTAAA |

FAM (Fluorescein); IABkFQ (Iowa Black Fluorescent Quencher); ZEN (Zen Fluorescent Quencher)


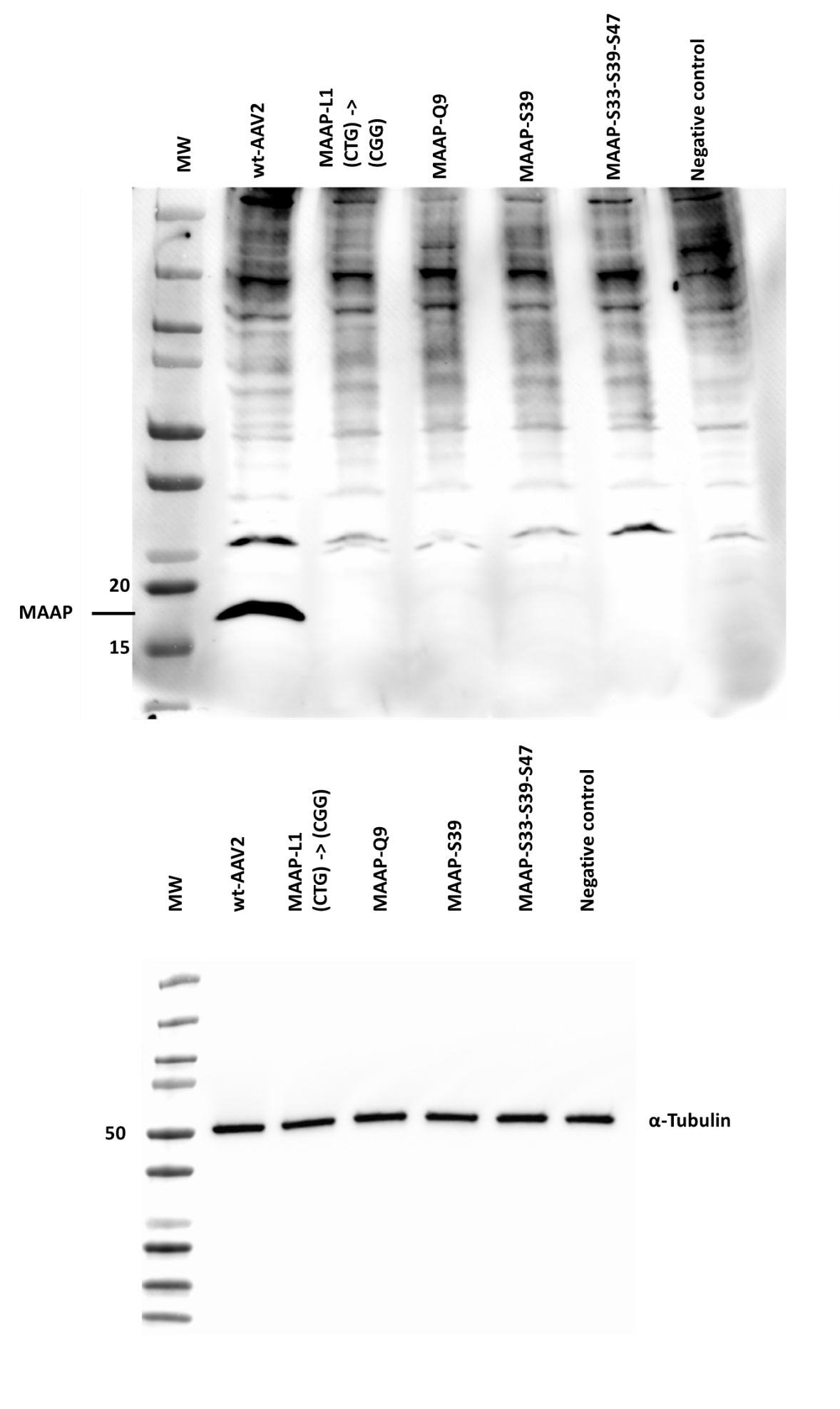


**Fig 2A. Original blots.**


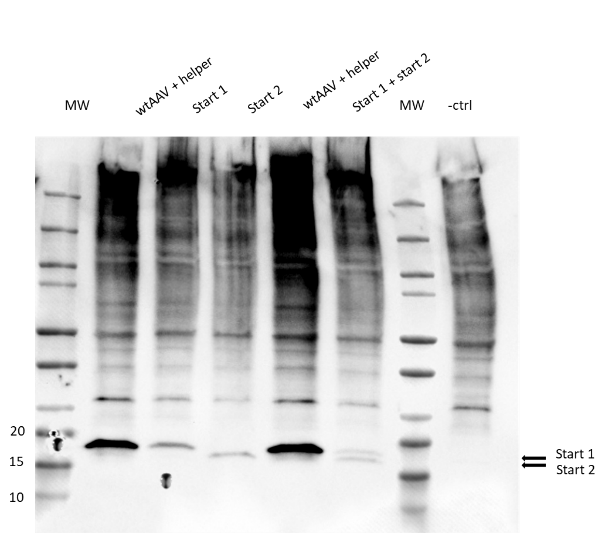


**Fig 2B. Original blot.**


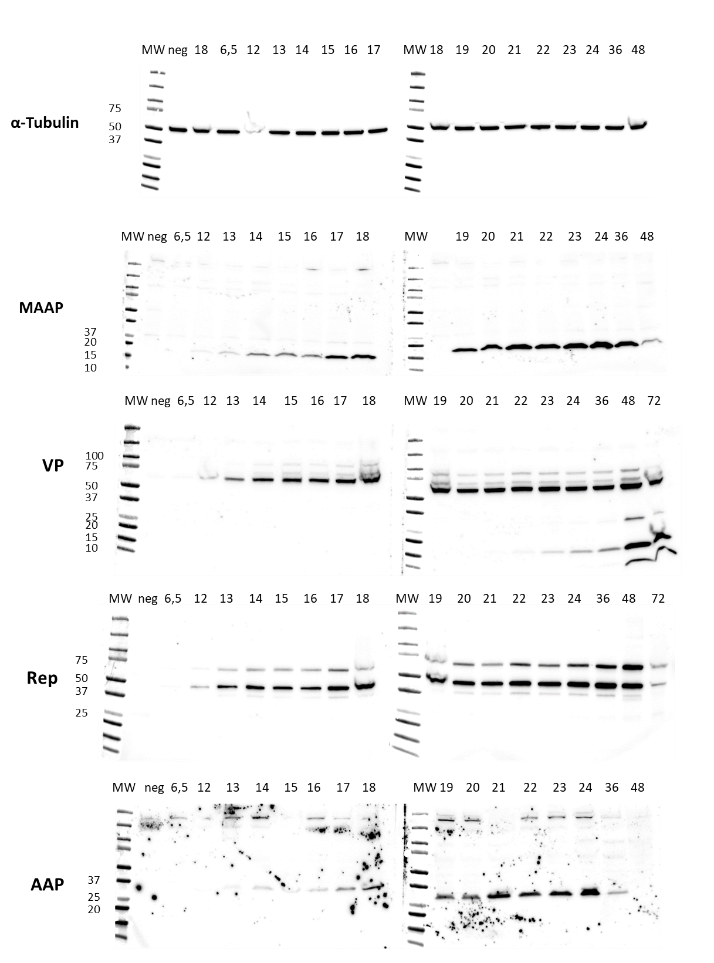


**Fig 3. Original blots.**


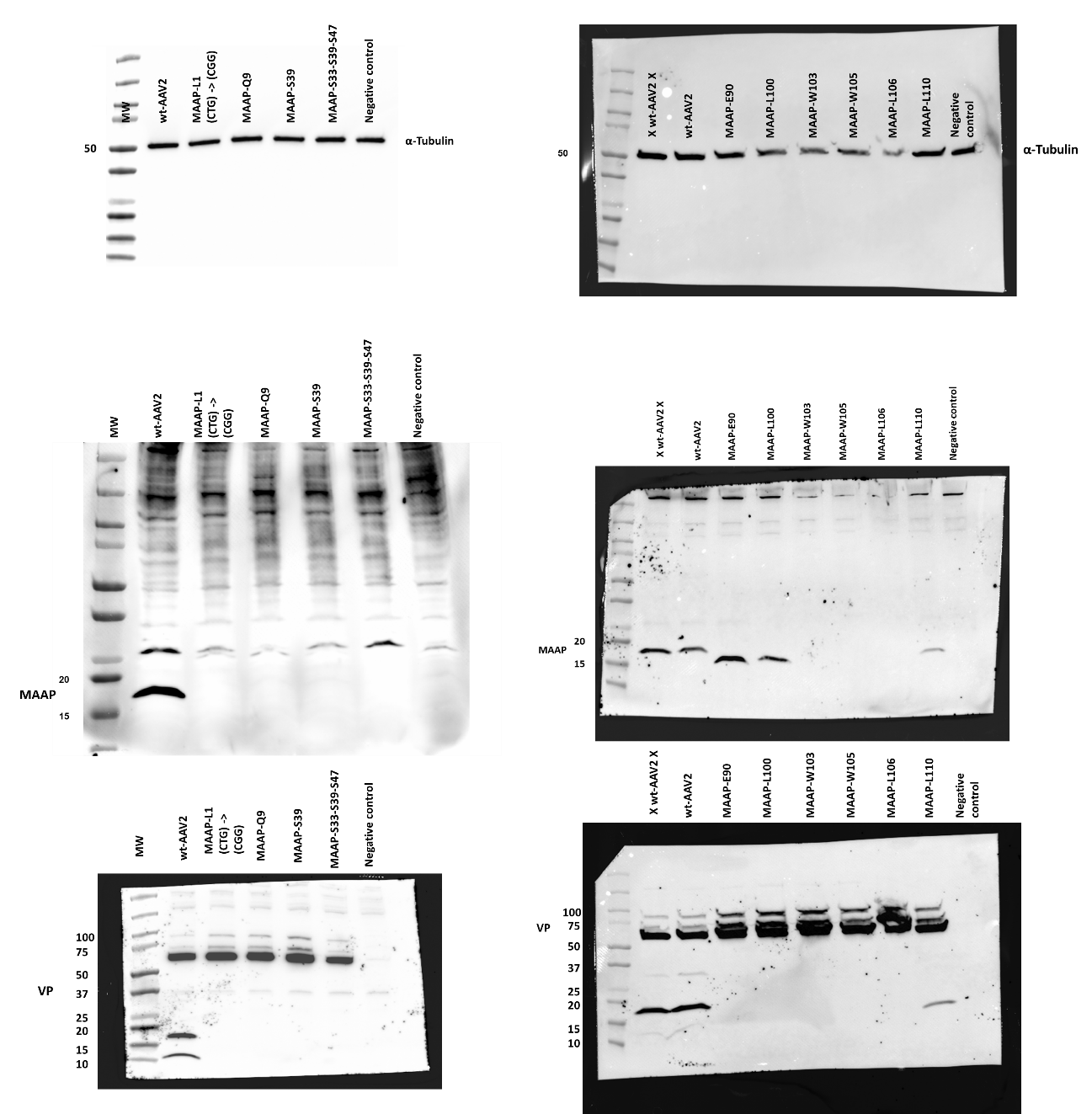


**Fig 7. Original blots, upper panel.**


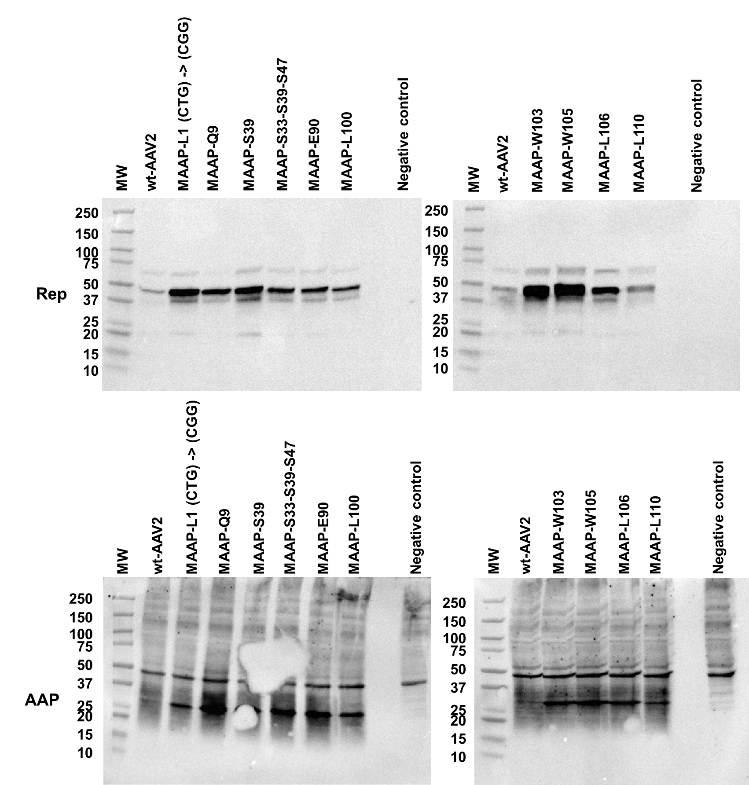


**Fig 7. Original blots, lower panel.**


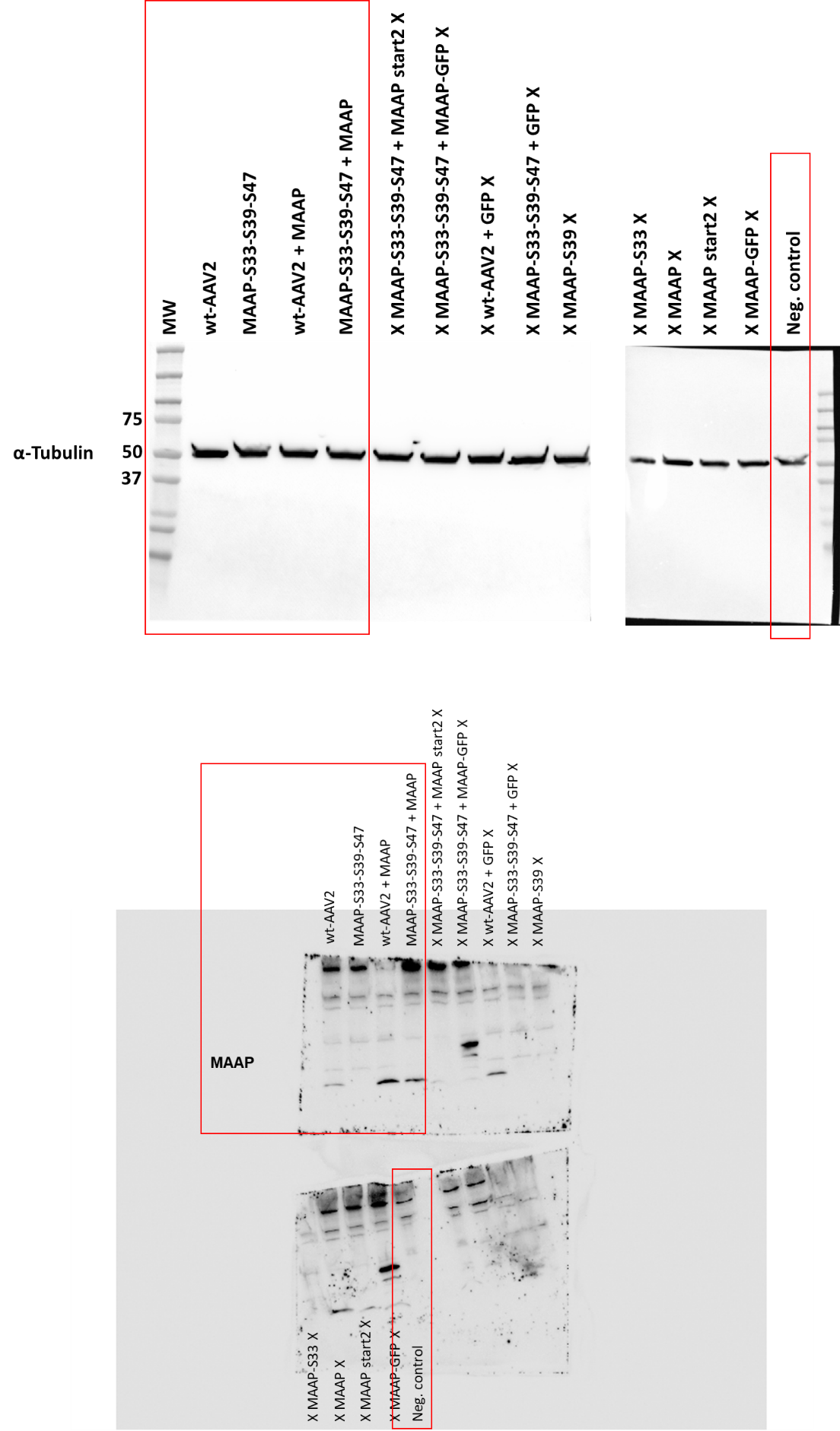


**S3 Fig. Original of α-Tubulin and MAAP**

Red boxes delimit the samples presented in S3 Fig.


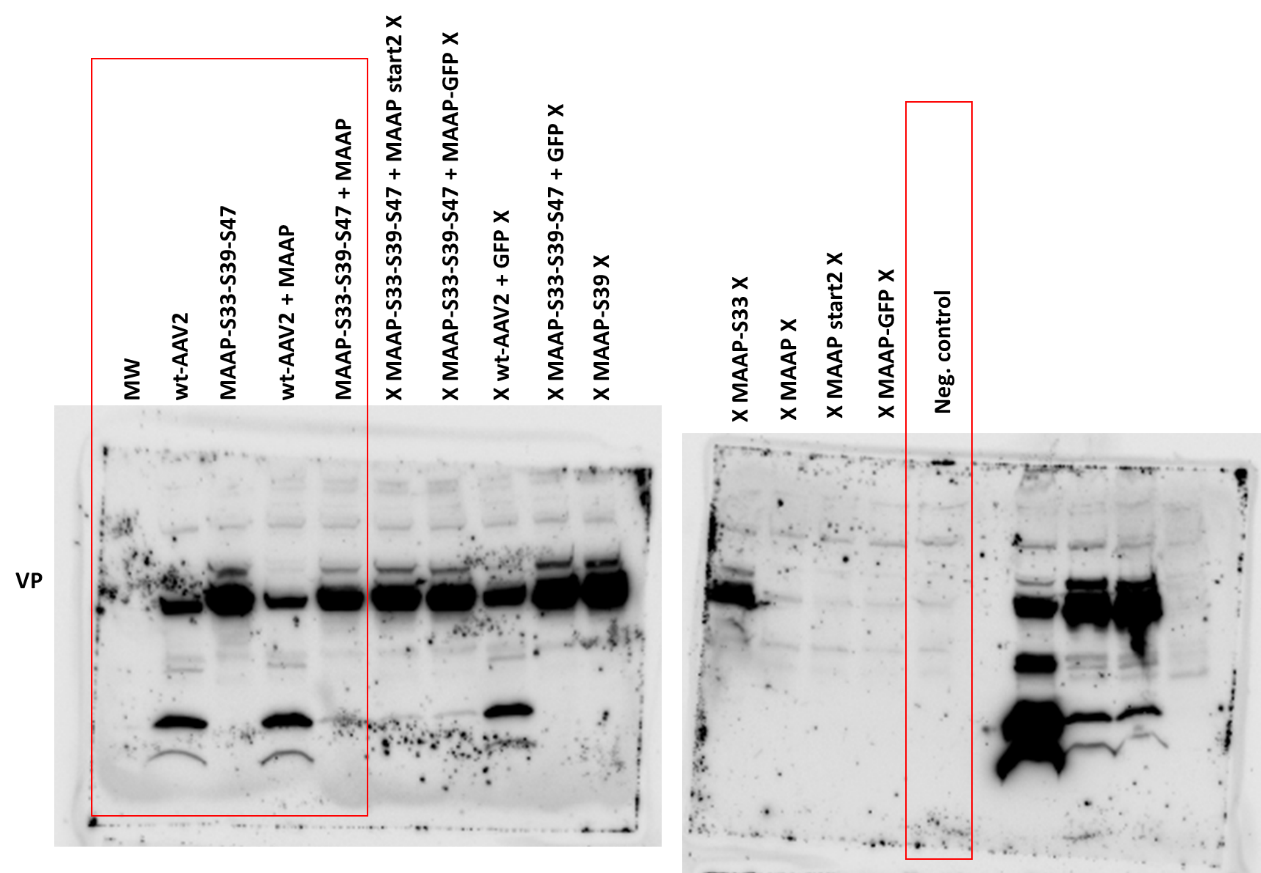


**S3 Fig. Original of VP**

Red boxes delimit the samples presented in S3 Fig.
